# A Genomewide Functional Network for the Laboratory Mouse

**DOI:** 10.1371/journal.pcbi.1000165

**Published:** 2008-09-26

**Authors:** Yuanfang Guan, Chad L. Myers, Rong Lu, Ihor R. Lemischka, Carol J. Bult, Olga G. Troyanskaya

**Affiliations:** 1 Lewis-Sigler Institute for Integrative Genomics, Carl Icahn Laboratory, Princeton University, Princeton, New Jersey, United States of America; 2 Department of Molecular Biology, Princeton University, Princeton, New Jersey, United States of America; 3 Department of Computer Science, Princeton University, Princeton, New Jersey, United States of America; 4 The Jackson Laboratory, Bar Harbor, Maine, United States of America; University of Chicago, United States of America

## Abstract

Establishing a functional network is invaluable to our understanding of gene function, pathways, and systems-level properties of an organism and can be a powerful resource in directing targeted experiments. In this study, we present a functional network for the laboratory mouse based on a Bayesian integration of diverse genetic and functional genomic data. The resulting network includes probabilistic functional linkages among 20,581 protein-coding genes. We show that this network can accurately predict novel functional assignments and network components and present experimental evidence for predictions related to Nanog homeobox (Nanog), a critical gene in mouse embryonic stem cell pluripotency. An analysis of the global topology of the mouse functional network reveals multiple biologically relevant systems-level features of the mouse proteome. Specifically, we identify the clustering coefficient as a critical characteristic of central modulators that affect diverse pathways as well as genes associated with different phenotype traits and diseases. In addition, a cross-species comparison of functional interactomes on a genomic scale revealed distinct functional characteristics of conserved neighborhoods as compared to subnetworks specific to higher organisms. Thus, our global functional network for the laboratory mouse provides the community with a key resource for discovering protein functions and novel pathway components as well as a tool for exploring systems-level topological and evolutionary features of cellular interactomes. To facilitate exploration of this network by the biomedical research community, we illustrate its application in function and disease gene discovery through an interactive, Web-based, publicly available interface at http://mouseNET.princeton.edu.

## Author Summary

Functionally related proteins interact in diverse ways to carry out biological processes, and each protein often participates in multiple pathways. Proteins are therefore organized into a complex network through which different functions of the cell are carried out. An accurate description of such a network is invaluable to our understanding of both the system-level features of a cell and those of an individual biological process. In this study, we used a probabilistic model to combine information from diverse genome-scale studies as well as individual investigations to generate a global functional network for mouse. Our analysis of the global topology of this network reveals biologically relevant systems-level characteristics of the mouse proteome, including conservation of functional neighborhoods and network features characteristic of known disease genes and key transcriptional regulators. We have made this network publicly available for search and dynamic exploration by researchers in the community. Our Web interface enables users to easily generate hypotheses regarding potential functional roles of uncharacterized proteins, investigate possible links between their proteins of interest and disease, and identify new players in specific biological processes.

## Introduction

Establishing a functional network is invaluable to furthering our understanding of gene function, pathways, and systems-level properties of an organism and can be a powerful resource in directing targeted experiments. The availability of diverse genome-scale data enables the prediction of networks encompassing all or at least most of the proteins in an organism. In *Saccharomyces cerevisiae*, probabilistic models have been used to predict the genomewide protein–protein functional interactions by integrating diverse data types [1]–[6]. Such probabilistic approaches have also been used in mammals to predict physical interactions [7],[8] and to generate expression networks [9]–[13]. In human, functional relationship networks have also been generated by integrating diverse interaction data [14]. However, it is still challenging to predict functional relationships through integrating diverse genomic data in mammalian model systems, due to the intrinsic complexity of these genomes and functional biases in individual datasets. Yet recent accumulation of both traditional targeted experiments, including protein physical interactions [15]–[17], gene-disease/phenotypic associations [18] and genome-scale data including gene expression and tissue localization [19]–[21], phylogenetic and phenotypic profiles [22],[23], as well as data retrieved based on homology [2],[24] provides the basis for establishing a global functional relationship network in the laboratory mouse [25].

**Figure 1:**
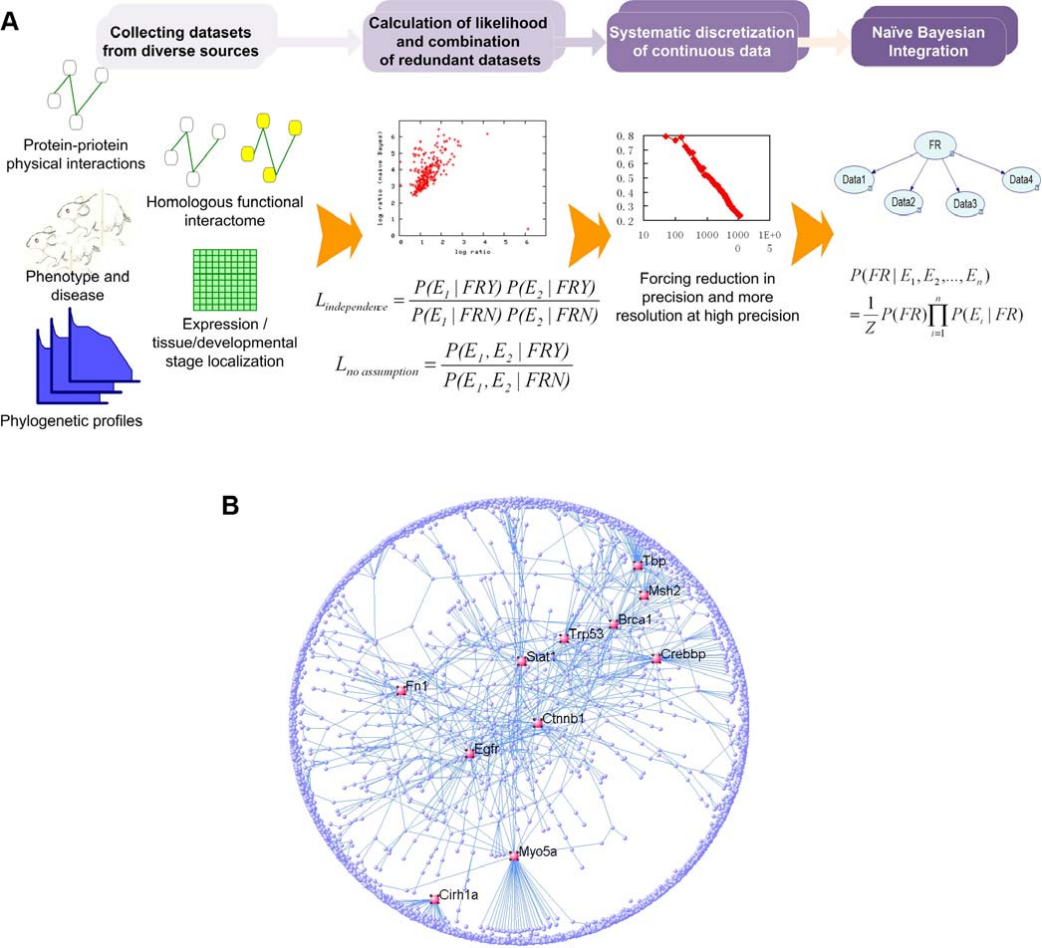
Strategy for processing and integration of diverse genomic data. (A) Schematic of the network integration pipeline. We collected five different types of data that are indicative of functional relationships, each of which may consist of multiple datasets (Table 1). We assessed the redundancy of each pair of datasets by comparing likelihood ratios with and without the independence assumption; datasets for which these values differed significantly were deemed mutually redundant and combined as a single input node in the Bayesian network for the purposes of integration. Finally, we systematically grouped continuous data and integrated all data with a naïve Bayes classifier to predict pair-wise functional relationships. (B) Global view of the predicted mouse functional network with higher than 0.8 confidence level of linkage. Nodes of high connectivity (more than 20 interactions) are labeled and highlighted in red.

We describe here a functional network in mouse generated by integrating a wide range of data types. In contrast to interactomes that represent physical interactions, our functional network predicts the probability that two proteins are involved in the same biological process and thus represents a more comprehensive combination of physical, genetic and regulatory linkages (Figure 1A). We demonstrate the utility of our network to predict gene functions and pathway components by both computational and experimental approaches. Further, we demonstrate how it can be used to further our understanding of the systems-level features of the mouse functional network. Our global functional network for the laboratory mouse is a valuable resource for analysis and annotation of the mouse proteome and can be used as a means of generating biological hypotheses for subsequent experimental validation, especially through the interactive public web interface available at http://mouseNET.princeton.edu.

## Results

### A Probabilistic Model To Predict Functional Relationships by Integrating Diverse Data Types

Bayesian networks have been used successfully for integrating diverse data sources in many biological settings, including protein function prediction [3],[6], prediction of genetic interactions [26], physical interactions [4],[7] and most relevant to this work, prediction of functional networks in *S. cerevisiae* [2],[5],[6] and human [14]. The Bayesian approach is especially well-suited to our problem, where many genome-scale data have missing values and collections of individual investigations may not be a complete representation of genome profiles. Based on a Bayesian framework, we designed a method that combines redundant datasets, processes continuous data, minimizes over-fitting and finally, integrates all experimental evidence (Table 1) in a confidence-based manner to estimate the genomewide pair-wise probabilities of functional linkage (Figure 1A). The resulting mouse interactome includes 20,581 genes, with edges representing the probability of functional relationship between each pair (Figure 1B). As demonstrated below, creation of this functional network through integrating diverse data sources can facilitate identification of novel pathway components and represents a powerful resource for understanding genetic diseases and network evolution.

**Table 1:** Data sources used for functional interactome integration.

Data Type	Data Sources	Date/Version	Number of Protein Pairs
**Protein–protein physical interaction data**	BIND [15]	01/21/07	2,709
	DIP [17]	01/21/07	47
	GRID [16]	10/16/06	8,144
	OPHID [24]	10/28/06	32,342
**Phenotype/disease**	MGI phenotype [18]	01/22/07	2,765,378
	OMIM disease	01/22/07	
**Phylogenetic profiles**	Inparanoid [23]	Version 4.0	123,284,253
	BioMart [22]	01/22/07	127,017,891
**Homologous functional relationship predictions**	bioPIXIE (yeast) [2]	10/30/06	4,280,332
**Expression and Tissue localization**	SAGE [19]	Version 02/14/06	139,871,175
	Zhang et al [34]	N/A	92,011,395
	Su et al. [42]	N/A	165,756,528

### MouseNET Recovers Functional Relationships

**Figure 2:**
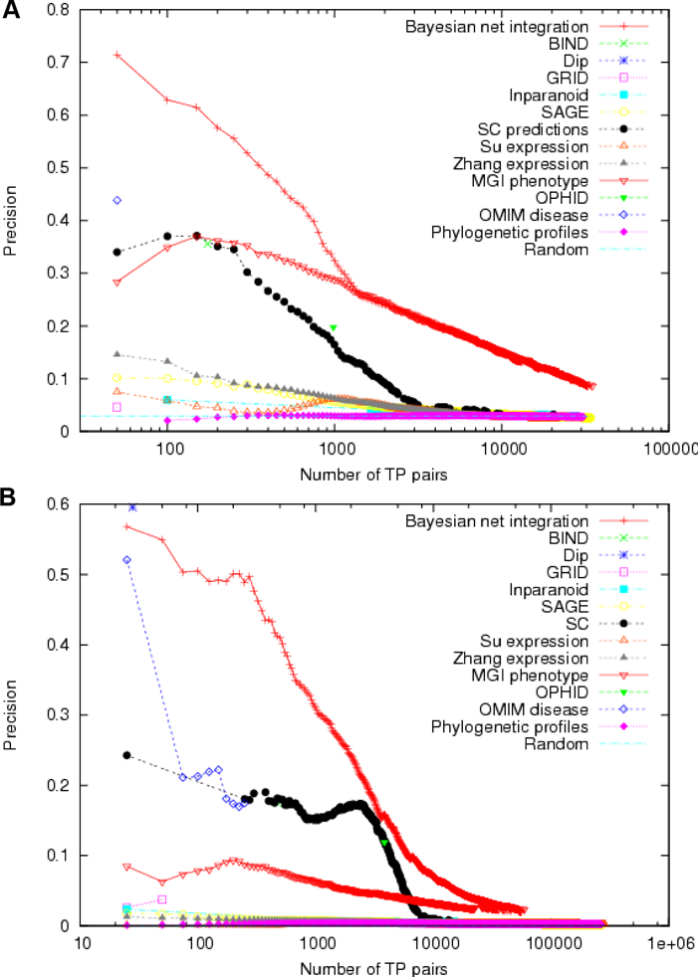
Computational performance analysis of the integrated network to predict functional relationships and the relative performance of different datasets. (A) Five-fold cross-validation of the integrated results applied to predict gold standard pairs defined by co-annotation to specific GO terms. Positive pairs were defined as those having at least one co-annotation to a specific GO term. Negative pairs are those that have a specific annotation, but share no co-annotations. Precision, or the fraction of correct predictions out of all predictions made, is measured across a number of cutoffs in prediction confidence (higher cutoff allows for less predictions of higher quality, and lowering the cutoff allows more predictions to be made at the cost of some decrease in accruacy). MouseNET predictions always have higher accuracy than those of the individual datasets. (B) Performance of the integrated results when evaluated against a different test set where positives are defined as pairs co-annotated to the same KEGG pathways, and negatives are pairs in which both members are annotated in KEGG, but share no co-annotations. Both performance measurements show that the integrated results are better in recovering known functional relationships than individual datasets.

A key application of a functional network prediction is to uncover novel pathway components. We first evaluated the accuracy of our predicted network through cross-validation analysis on known functional linkages (co-annotations of proteins to specific Gene Ontology [27] terms), which is the standard for unbiased computational evaluation. In short, cross-validation can be used to assess the accuracy of predictions by evaluating the system's accuracy in recovering subsets of known annotations withheld during the training process. Our integrated network is substantially more successful in predicting known functional linkages than any of the individual datasets and making more correct predictions (demonstrating higher precision) at every confidence cutoff (Figure 2A). This result is robust to using a different annotation standard, i.e., co-annotation to the same Kyoto Encyclopedia of Genes and Genomes [28] (KEGG) pathways (Figure 2B). Notably, although the relative performance of datasets varies with different standards, the consistently good performance of our results suggests that the integrated predictions are robust to variations in the annotation standard.

A common pitfall of many global integration schemes is the tendency to make precise predictions over only a limited set of biological processes [29]. Thus we evaluated the functional composition of our integrated results using KEGG, which is an accurate representation of our current knowledge of different pathways. The integrated network exhibits a balanced representation of a large group of pathways, even though many individual datasets have significant functional biases (Figure S1, the complete statistics of this functional composition analysis are included in the Dataset S1). For instance, the protein–protein interaction data obtained from the Biomolecular Interaction Network Database (BIND) [15] is significantly skewed towards the processes of focal adhesion. In contrast, given the broad functional coverage of the integrated network, we expect our approach will be useful in further characterization of a variety of pathways.

### MouseNET Predicts Novel Pathway Components and Gene Functions

**Figure 3:**
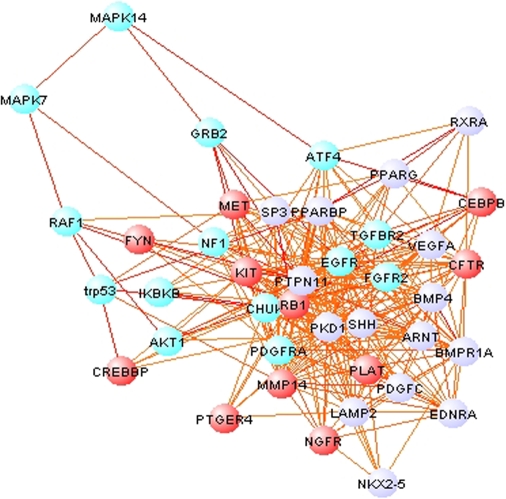
Analysis of MAPK pathway predictions based on the integrated functional network. Predictions were derived by iteratively sampling 10 proteins from the known MAPK pathway and finding the closest 40 neighbors based on network adjacency. The results shown are based on an aggregation of 300 such samplings. Bright blue denotes proteins annotated to the canonical MAPK pathway in KEGG. Many of the newly predicted components, although not annotated in KEGG, are supported in the literature (Table S2) and are colored in red. Predictions without literature support are colored in purple. Linkages predicted to be above 0.5 confidence level by our integrated network are shown.

The high accuracy in predicting co-annotation to KEGG pathways (Figure 2B) by our network and its broad functional coverage (Figure S1) suggest that mouseNET can accurately capture pathway-based functional linkages for a variety of processes. We thus focused specifically on the predicted functional network for the major conserved signaling pathways related to development, including Hedgehog, Wnt, MAPK, TGF-β, Notch, and Toll-like receptor signaling pathways. We find that in addition to recovering known pathway components (Figure S2), these networks include a number of proteins not previously annotated to the pathway. Many of these novel predictions have reasonable experimental support in the literature. For example, in the 40 most tightly connected nodes surrounding known MAPK pathway proteins (Figure 3), 14 of them are annotated as the canonical pathway components in KEGG (*p*<10^−10^, hypergeometric distribution). Furthermore, two of the other nodes (*Kit*, MGI:96677 and *Shh*, MGI:98297) are not annotated to the MAPK pathway in KEGG but are annotated in the Gene Ontology [27] to be MAPK-related. Another nine unannotated predictions in the cluster of 40 have been suggested in literature to be involved in the MAPK pathway (Table S2 and Text S1). Thus, our system not only recovers well-established knowledge but also implicates novel pathway components, and therefore could be a powerful tool for generating hypotheses for experimental approaches.

Our genomewide prediction of protein function based on the integrated network produced 689 novel annotations with an estimated 80% precision. A subset of these new predictions was evaluated through examination of the literature by MGD curators and the precision estimate was confirmed (Dataset S2). Of these, 17 predictions were confirmed based on literature evidence at the level sufficient for annotation in MGI, and another six were found to have some support in the literature, but at a level not yet sufficient for GO annotation. For example, *Retn* (MGI:1888506), which does not have a GO biological process or KEGG pathway annotation, was predicted with high confidence (over 0.8) to be involved in glucose homeostasis (GO:0042593). The loss of *Retn* was indeed found to improve glucose homeostasis in leptin deficiency [30], confirming the prediction. This evaluation demonstrates that through integrating information from diverse sources, the system is capable of making accurate novel predictions on genes not previously annotated in GO or KEGG.

### Experimental Validation by Nanog Down-Regulation Induced Cell Differentiation

**Figure 4:**
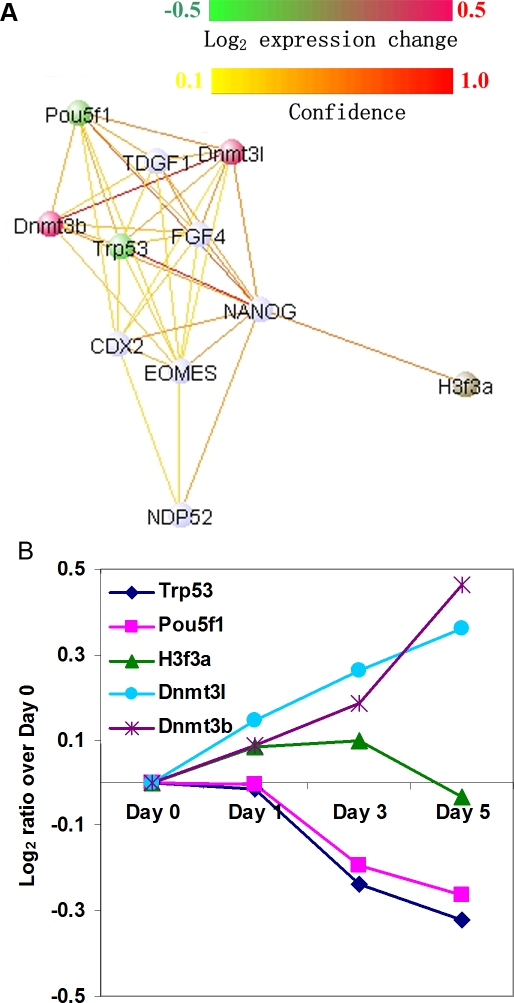
Validation by *Nanog* down-regulation experiment. (A) The top 10 neighbors of Nanog as predicted by Bayesian integration. Links with more than 0.1 confidence level are presented in the figure. The colors of *Trp53*, *Dnmt3b*, *Dnmt3l*, *Pou5f1*, and *H3f3a* indicate the Log_2_ changes in protein expression on the fifth day after Nanog knock down compared to day 0. (B) Protein expression changes detected by mass spectrometry after Nanog knock-down. Four of the five top neighbors detected in the nucleus have significant changes in protein expression level, with increasing changes during the time course.

To further validate novel functional relationships predicted by our integrative network, we investigated proteins predicted to cluster around the homeobox transcription factor *Nanog* (MGI:1919200), which is an essential gene responsible for maintaining embryonic cell fate. Specifically, we experimentally down-regulated the expression of *Nanog*, and observed the nuclear protein expression changes of the top functional interactors in our predicted network by mass spectrometry. Five of the top 10 *Nanog* interactors predicted by mouseNET (Figure 4A) were detected in the nuclei and thus, we could evaluate their expression following *Nanog* down-regulation. We observed that after *Nanog* down-regulation, expression levels of four of them either significantly increased (DNA (cytosine-5-)-methyltransferase 3-like, *Dnmt3l*, MGI:1859287 and DNA methyltransferase 3B, *Dnmt3b*, MGI:1261819) or decreased (transformation related protein 53, *Trp53*, MGI:98834 and POU domain, class 5, transcription factor 1, *Pou5f1*, MGI:101893) (*p*<0.1 when compared to the overall distribution of the nucleus-detected proteins, Figure S8). Of those, *Pou5f1* has also been previously shown to be involved in ES cell regulation [31],[32] and it has significant overlap in genomic binding targets with *Nanog* [33],[34]. Furthermore, the change in expression for these four proteins is consistent for different time points after *Nanog* knock-down, and increases consistently over the time course (Figure 4B). This experimental verification demonstrates that our system is a powerful tool which can aid researchers in generating accurate hypotheses for discovery of proteins involved in a specific cellular process.

Our functional network can also highlight information about physical interactions and transcriptional binding sites. For example, the 17 physical interactions with *Nanog* identified by Wang et al. were highly enriched in pairs of high functional relationship confidence (Mann-Whitney U test *p* = 0.00069). In addition, on the transcription level, the *Nanog* binding loci associated genes [34] were also highly enriched in high confidence functional interactors of *Nanog* predicted by our network (U test *p* = 3.98E-18). Therefore, by integrating a diverse collection of data, mouseNET enables users to explore variety types of functional associations, including physical interactions and transcriptional level regulation.

### Topological Analysis Reveals Distinct Characteristics of Modulators of Diverse Processes

**Figure 5:**
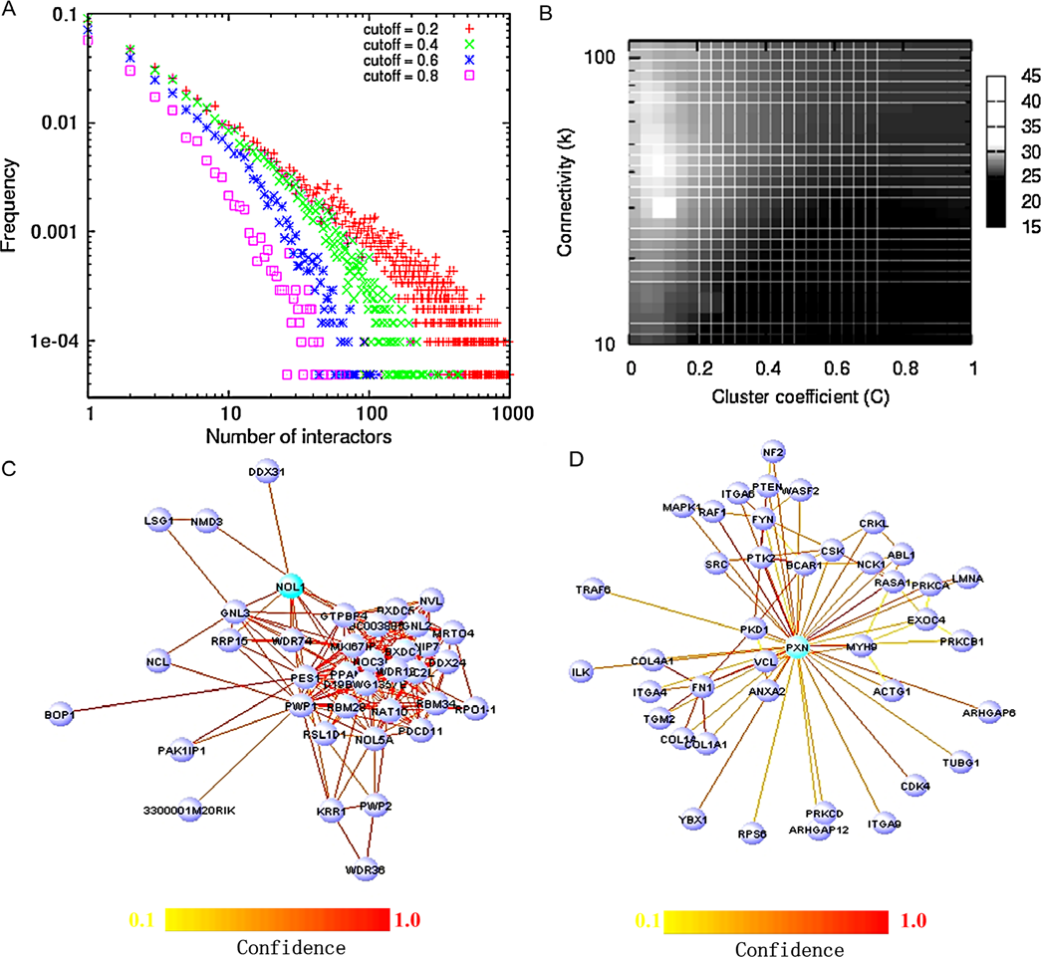
Topological properties of the functional network. (A) The degree (node connectivity) distribution of the integrated functional network (log_10_ scale) for several different edge probability cutoffs. (B) Connectivity (at 0.6 cutoff in confidence) versus clustering coefficient. The color represents the number of processes represented in that gene's local network (top 40 neighbors). At the same level of connectivity, proteins with smaller clustering coefficients tend to participate in more processes. Local networks centered around *Nol1* (C) and *Pxn* (D). While both genes have roughly equivalent node degree (∼50 confident connections), a potential modulator of multiple pathways (D), however, is differentiated from other hub genes (such as (C)) in that it has a lower clustering coefficient and thus the network centered at *Pxn* is less densely connected.

MouseNET provides a valuable resource to characterize the systems-level features of a model organism, which is a critical issue in understanding the organization and dynamics of the proteome. In the mouseNET network, the majority of proteins have only a small number of connections (Figure 5A), yet the presence of a few highly connected nodes (Figure 1B) implies central modifiers of the proteome. These ‘hub’ genes (at confidence cutoff 0.6) are enriched in regulation of response to stress, DNA metabolic process and cell cycle, (Bonferroni-corrected *p*<1.0E-9) (Table 2). Additionally, these hubs were significantly enriched (Bonferroni-corrected *p* = 8.3E-10) for ‘chromosome organization and biogenesis’, which is in agreement with a previous study in *C. elegans* that identified a class of genetic interaction hubs, all six of which were chromatin regulators [35].

**Table 2:** GO SLIM (Biological Process) enrichment of potential modulators of several pathways (*C_i_*<0.15, *N*≥10) and highly connected genes (*N*≥10).

GO Term	GO Term Name	*C_i_*<0.15, *N*≥10	*N*≥10
GO:0000003	Reproduction	1.12E-04	1.88E-08
GO:0016043	Cell organization and biogenesis	<1.01E-12	<1.01E-12
GO:0016265	Death	<1.01E-12	2.66E-11
GO:0006950	Response to stress	<1.01E-12	<1.01E-12
GO:0009628	Response to abiotic stimulus	7.63E-07	1.15E-09
GO:0006259	DNA metabolic process	2.93E-11	<1.01E-12
GO:0008283	Cell proliferation	<1.01E-12	<1.01E-12
GO:0044238	Primary metabolic process	<1.01E-12	<1.01E-12
GO:0015031	Protein transport	1.22E-06	<1.01E-12
GO:0006810	Transport	1.53E-01	6.32E-06
GO:0009605	Response to external stimulus	3.45E-10	5.52E-05
GO:0009653	Anatomical structure morphogenesis	<1.01E-12	7.52E-14
GO:0007165	Signal transduction	<1.01E-12	5.34E-01
GO:0008152	Metabolic process	<1.01E-12	<1.01E-12
GO:0030154	Cell differentiation	<1.01E-12	<1.01E-12
GO:0050789	Regulation of biological process	<1.01E-12	<1.01E-12
GO:0007267	Cell-cell signaling	1.40E-05	2.82E-03
GO:0007154	Cell communication	<1.01E-12	3.27E-01
GO:0008219	Cell death	<1.01E-12	2.66E-11
GO:0006139	Nucleobase, nucleoside, nucleotide and Nucleic acid metabolic process	1.76E-11	<1.01E-12
GO:0006996	Organelle organization and biogenesis	4.00E-08	<1.01E-12
GO:0009719	Response to endogenous stimulus	1.60E-09	<1.01E-12
GO:0006464	Protein modification	<1.01E-12	2.29E-08
GO:0006350	Transcription	5.48E-11	7.69E-09
GO:0007275	Multicellular organismal development	<1.01E-12	<1.01E-12
GO:0019538	Protein metabolic process	<1.01E-12	<1.01E-12
GO:0006412	Translation	3.12E-02	1.78E-10
GO:0007010	Cytoskeleton organization and biogenesis	2.83E-06	2.29E-11
GO:0009790	Embryonic development	<1.01E-12	1.98E-13
GO:0040007	Growth	1.08E-12	8.69E-09
GO:0007049	Cell cycle	<1.01E-12	<1.01E-12
GO:0000003	Reproduction	0.000112338	1.88E-08

We further analyzed the topology of the functional network surrounding these hubs and found distinct characteristics that correlate with their role in the cell. Proteins with high connectivity may appear in densely connected modules, or alternatively, they could be linkers of multiple functional modules and participate in several pathways [36]. To investigate these two classes, for each gene we computed the clustering coefficient, *C*, which gives the probability that its interactors are connected to each other. We found that low clustering coefficients, when controlled for node degree, are critical indicators of proteins participating in more biological pathways (Figure 5B). This trend is robust against different confidence cutoff levels for the interactions (Figure S3). For example, both nucleolar protein 1 (*Nol1*, MGI:107891) and paxillin (*Pxn*, MGI:108295) have 50 functional linkages with more than 0.6 confidence in interactions (Figure 5C and 5D). However, the former, which has a *C* of 0.44, is involved in only the rRNA processing pathway, while the latter, with a *C* of 0.06, is known to be involved in multiple biological processes, including activation of MAPK activity, branching morphogenesis of a tube, cell adhesion and protein folding. Furthermore, we found that the set of proteins with low clustering coefficients, but not the set of all proteins with only high node degree, is highly enriched for ‘signal transduction’ (Table 2), probably because proteins involved in signal transduction are central to cross-talk among multiple pathways and the cell's diverse response to various stimuli. Thus, the topology of the functional network contains important clues to the global organization of the proteome; and in addition to connectivity, we demonstrate that the clustering coefficient is a critical factor characterizing modifiers of multiple biological pathways.

### Phenotypic and Disease Effects in Relation to Topology and Functional Participation

Global modeling of functional linkages provides a general framework to analyze the relationship between local network properties and functional consequences of individual gene perturbations. For example, previous studies have predicted that the network connectivity is correlated with the propensity of a protein to be essential [37],[38]. Recently, however, there has been debate over whether this relationship is indeed true in yeast or human [39],[40], the main issue being whether high connectivity is truly a property of the underlying network or simply an effect of intense study of the essential gene set (i.e., annotation or investigational bias).

**Figure 6:**
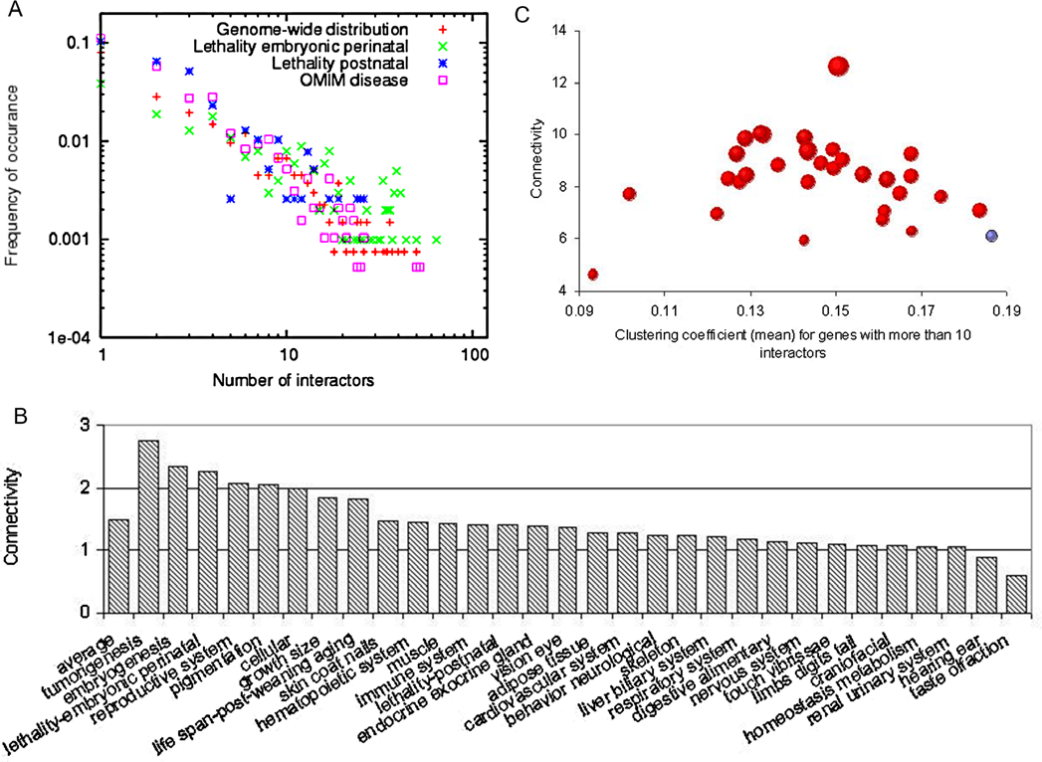
Relationship between phenotypic effects and local network configuration. (A) Comparison of connectivity (at 0.6 confidence) between essential and non-essential genes, and between genes whose orthologous mutants cause disease in human and those with no apparent phenotype. Both comparisons are based on a functional network excluding any phenotypic or disease input data to avoid circularity, and excluding any datasets involving individual investigation results to avoid investigational biases. (B) The average number of functional interactions (at 0.6 confidence) for genes within each phenotypic class. (C) Based on a functional network from integration of all available data, the clustering coefficient is consistently lower for genes having diverse categories of phenotypes; the size of the bubble is proportional to the number of processes represented in nearest neighbors (40 closest proteins). This trend holds true in a network where all individual investigations are excluded, suggesting this trend is not an effect of investigational bias.

To address this question in the mouse functional network and control for investigation bias, we constructed two networks: one including all input data except knock-out phenotype information, and one including only whole-genome datasets. To avoid the caveat that not all gene knock-outs have been constructed, only genes that have been knocked out or targeted were included in all statistical analyses. For the first functional network, essential genes or disease-associated genes are significantly more connected than average (*p*<10^−18^ for perinatal lethality, *p*<10^−9^ for postnatal lethality, and *p*<10^−6^ for disease-associated genes, Mann-Whitney U test) (Figure S4A). However, in the functional network based on only whole-genome datasets, the difference between essential and non-essential sets was not significant, nor was that between disease-related set and the genome average (Figure 6A), suggesting the observed relationships between essentiality and network connectivity are likely to be explained by investigational biases in our case. This result is consistent with a previous study [41] which suggested that the vast majority of disease genes show no tendency to encode physical interaction hubs in human data. We further considered whether connectivity and local topology in our functional network relate to other perturbation phenotypes. Although most phenotype-responsible gene groups (Table S1) have a higher than average connectivity based on all available input data (Figure S4B), only proteins involved in tumorigenesis, embryogenesis still have significantly higher connectivity than average (*p*<0.05) on the whole-genome-data-only network (Figure 6B). This result highlights that the variation in intensity of study for genes can cause significant biases in the conclusions reached when comparing the connectivity of different groups of genes.

We observed that all groups of phenotype-associated genes have a lower clustering coefficient than average, and most participate in more biological pathways (Figure 6C). This conclusion holds true when controlling for investigational biases. For example, *Trp53*, with very high connectivity (Figure 1B) and particularly low clustering coefficient (0.02252), is essential during both embryonic perinatal and postnatal stages and plays a role in tumorigenesis, the reproductive system, and has ten other high level phenotypes (Table S1) according to the Mouse Genome Informatics (MGI) database [18]. This result implies that hubs with low clustering coefficient and participating in multiple pathways are important buffers of the genome, and that mutations or other disruptions of these genes are likely to be related to a detrimental phenotypes and, likely, disease.

### Comparison of Yeast and Mouse Functional Networks

**Figure 7:**
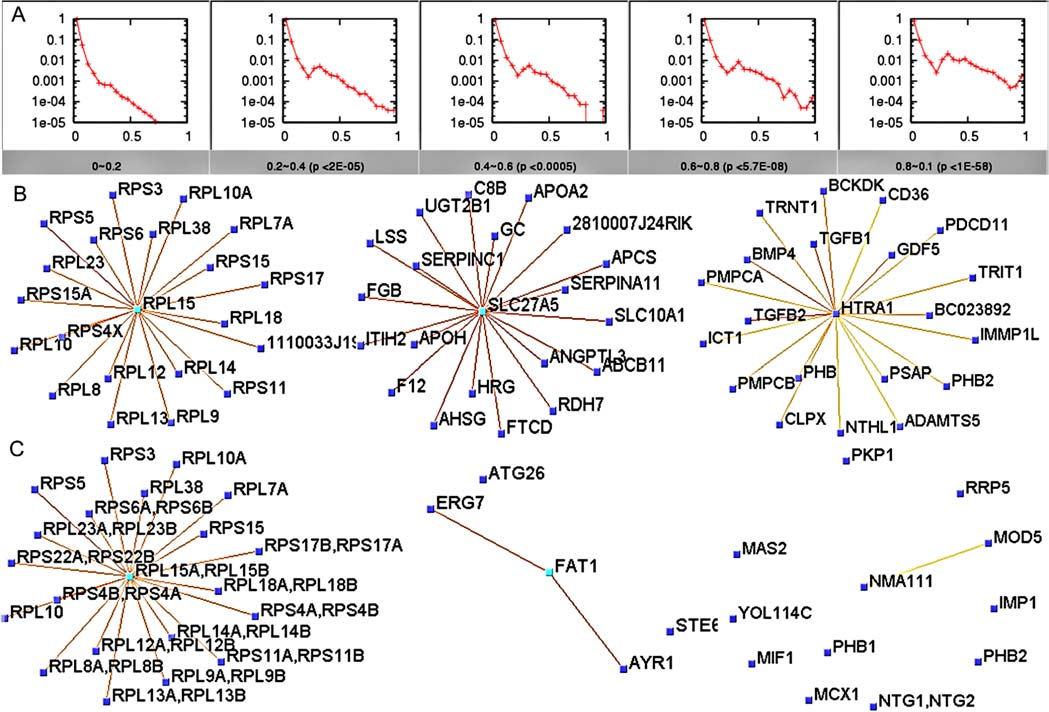
Comparison of yeast and mouse interactome and identification of mouse-specific functional linkages. (A) Distribution of functional relationships in mouse for the corresponding interaction between orthologous genes in yeast. For each graph, the range of edge confidences in the yeast network is labeled below, and relative frequency (*y*-axis) is plotted against confidence of functional relationships for orthologous pairs in mouse. The *p*-value (Mann-Whitney U test) for each sub-figure indicates the significance of the difference between the distribution of mouse functional relationships in that bin and relationships in the range of 0.0–0.2 yeast interaction confidence (the first graph). (B) Subgraphs of mouse interactome centered at *Rpl15* (MGI:1913730), ribosomal protein L15; *Slc27a5* (MGI:1347100): solute carrier family 27 (fatty acid transporter), member 5; *Htra1* (MGI:1929076): HtrA serine peptidase 1. (C) To visualize how interactions in mouse were evolutionarily acquired, we adapted a method of collapsing paralogous genes [47] in the yeast interactome. Yeast orthologs of mouse genes in (B) appear at the same positions in (C). The links represent the average weight of the interactions between paralogs.

Genome evolution on the sequence level has been studied intensively during the past decades. Studies of functional evolution on the genome-scale, on the other hand, require comprehensive profiling of proteins, which is difficult due to largely incomplete annotation of protein function in most organisms. Here, we demonstrate that mouseNET is a valuable resource for cross-species functional evolution studies by comparing it to the *S. cerevisiae* network [2]. To avoid circularity caused by integration of sequence similarity information, we generated a functional network that excludes all orthology-based input data. Given these mouse and yeast networks, we first investigated whether functional linkages are conserved between pairs of orthologs as identified through InParanoid [23]. Our results indicate that high-confidence functional linkages in *S. cerevisiae* are strongly predictive of functional linkages between orthologous gene pairs in mouse (Figure 7A for statistical analysis).

We also investigated the conservation of functional neighborhoods in the mouse and yeast networks. To make the datasets comparable, we included only orthologous pairs in the conservation statistical analysis. We found that the two networks vary from a high degree of conservation to almost no conservation (Figure 7B and 7C). Functional linkages between proteins involved in response to stress, response to endogenous stimulus, catabolic process, DNA metabolism, cell cycle, and other core biological processes and components were highly conserved between yeast and mouse (Table 3), e.g., the ribosomal protein L15 (*Rpl15*, MGI:1913730; Figure 7B and 7C). In contrast, functional relationships in processes specific to higher organisms, including, behavior, embryonic development, multicellular organismal development and anatomical structure morphogenesis were limited to the mouse network (Table 4). For example, the HtrA serine peptidase 1 (*Htra1*, MGI:1929076) plays a role in BMP signaling pathway [42], but its ortholog in yeast, YNL123W (*Nma111*, SGD: S000005067) is involved in apoptosis and lipid metabolic process [43],[44] (Figure 7B and 7C). The newly generated interactions for these mouse-specific functional networks originated through a combination of orthologous pairs in yeast and novel connections with existing genes or genes that have no ortholog in yeast (Figure 7B and 7C). Interestingly, ion transport was among the list of enriched processes for both conserved and unconserved subgraphs. We found that in conserved subgraphs, these genes were enriched in energy-coupled proton transport, which is conserved from yeast to mammals. In contrast, in the unconserved subgraphs, this enrichment of ion transport was due to genes involved in metal-ion or chloride transport, probably because of their involvement in the neural system. Details regarding the enrichment statistics are available in the Dataset S3.

**Table 3:** Conservation between yeast and mouse functional relationships.

GO Term	GO Term Name	Bonferroni-Corrected *p* Value
GO:0006950	Response to stress	2.27E-09
GO:0009719	Response to endogenous stimulus	2.36E-09
GO:0009056	Catabolic process	1.37E-08
GO:0006259	DNA metabolic process	2.26E-08
GO:0007049	Cell cycle	1.01E-05
GO:0006091	Generation of precursor metabolites and energy	0.00266
GO:0006811	Ion transport	0.00600

GO SLIM (Biological Process) enrichment in mouse for genes of conserved interactions (higher than 0.6 confidence of functional relationship in both *S. cerevisiae* and in mouse) against all orthologous genes.

**Table 4:** Divergence between yeast and mouse functional relationships.

GO Term	GO Term Name	Bonferroni-Corrected *p* Value
GO:0015031	Protein transport	1.29E-05
GO:0006811	Ion transport	<E-06
GO:0005975	Carbohydrate metabolic process	<E-06
GO:0009607	Response to biotic stimulus	<E-06
GO:0006519	Amino acid and derivative metabolic process	<E-06
GO:0009628	Response to abiotic stimulus	<E-06
GO:0006464	Protein modification	<E-06
GO:0007275	Multicellular organismal development	<E-06
GO:0007165	Signal transduction	<E-06
GO:0007610	Behavior	<E-06
GO:0009653	Anatomical structure morphogenesis	<E-06
GO:0050789	Regulation of biological process	<E-06
GO:0007010	Cytoskeleton organization and biogenesis	<E-06
GO:0007154	Cell communication	6.15E-07
GO:0006810	Transport	1.34E-06
GO:0009790	Embryonic development	3.67E-05

GO SLIM (Biological Process) enrichment in mouse for genes whose interactions are not conserved from yeast (higher than 0.6 confidence of functional relationship in *S. cerevisiae* but less than prior in mouse) against all orthologous genes.

Comparative analysis of interactomes between species, such as that presented above, is no doubt a promising approach for answering a number of fundamental biological questions [45]. Previous studies, e.g., [40], have demonstrated the sparsity of our current knowledge of physical interactions in many organisms, which has led to a very limited set of identified conserved interactions. As demonstrated here, the comparison of higher-coverage functional networks based on probabilistic models for integrating diverse genomic data provide an alternative solution for studying the evolution of functional linkages between proteins.

### Example Application of the MouseNET Web Interface

#### Generating hypotheses for biological functions for a protein of interest based on integrating diverse data sources

An important application of the network analysis is to identify, for a protein of interest, which biological processes and pathways it participates in. Here, we use the mouseNET online query system to identify two different biological processes involving *Ace* (angiotensin I converting enzyme 1, MGI:87874). *Ace* is currently only annotated to metabolic process (GO:0008125) and proteolysis (GO:0006508) biological process terms in the Gene Ontology. *Ace* has a well-established central role in blood pressure regulation, evidenced by knock-out phenotypes [46], but it currently lacks annotation to the corresponding GO term. When mouseNET is queried with ‘*Ace*’, the system indeed suggests that the local network is highly enriched in blood pressure regulation (GO:0008217, *p* = 8.17E-4), including four proteins annotated directly to this term (*Agtr1a*, *Agtr1b*, *Ren1*, and *Agt*) (Figure S5A). The functional links between *Ace* and these four genes cannot be confidently surmised from any single input dataset; instead, they are supported by a combination of data from InParanoid [47], phenotype [48], OMIM [24], SAGE [19], and Zhang [21] expression data, indicating the important role of data integration for suggesting accurate functional role for proteins.

In the *Ace* predicted functional network, we also found enrichment for another unrelated process: menstrual cycle phase (GO:0022601), which currently is synonymous to estrous cycle in mouse GO annotation. Three of the top 40 interactors (*Stat5a*, *Nos3* and *Agt*) were annotated to this term (*p* = 3.73E-2), with support from InParanoid [23], phenotype [48], OMIM, SAGE [19], Su [20], and Zhang [21] expression data. Indeed, the expression cycle of *Ace* shown by immunohistochemistry is correlated with menstrual cycle in human [49], suggesting that mouseNET's prediction of *Ace* participation in the estrous cycle phase process is likely correct. This annotation is missing from existing annotation databases and such prediction would not be made based on genome scale pair-wise physical interaction studies. Because our system integrates diverse data sources and presents them in a network context, it can quickly allow biology researchers to reveal multiple independent roles of a single gene. mouseNET can thus serve both as a source of functional information for genes that have been previously investigated, but not yet annotated in public databases, as well as a method for directing experiments by hypothesizing novel roles for previously uncharacterized proteins.

#### Identifying disease-related genes through multiple queries of the mouseNET network

Because genes responsible for the same disease are often involved in related pathways, mouseNET provides a valuable resource for identifying novel disease gene candidates though its multiple-query feature. For example, by searching mouseNET with a set of genes (*Mapt*, *Sncaip*, *Tbp*, *Drd4*, *Ndufv2* and *Nr4a2*) already known to be involved in Parkinson's disease, we are able to extract other genes annotated to this disease and some novel candidates (Figure S5B). The top three interactors returned by mouseNET (*Uchl1*, *Dbh* and *Snca*) are already labeled with Parkinson's disease in OMIM, indicating the ability of our system to accurately identify other disease genes given some known ones. The fourth gene *Msx1* (Homeo box, msh-like 1) is not yet annotated to Parkinson's disease. However, its connection to several query genes (*Tbp* and *Mapt*) and to several proteins functionally related to the query set (*Mdm2*, *Fyn*, *Psen1*, *Apoe*, *Uchl1*, and *Dbh*) in mouseNET suggests its potential role in Parkinson's disease. Interestingly, *Msx1* was found to act as an intrinsic dopamine-neuron determinant during development, and therefore is very likely to be a candidate involved in Parkinson's disease, which leads to mesencephalic dopamine neuron degeneration. In addition, among the top three interactors, experiment using transgenic mice shows that *Uchl1* mutant could lead to dopaminergic neuronal loss [50]; *Dbh* is a critical gene involved in dopamine biosynthesis; and *Snca* has been suggested to be an essential regulator of dopamine neurotransmission [51]. Notably, query of *Tbp* alone results in a list of transcription-related genes that has no significance with the particular disease. The novel candidate *Msx1* is only identified with multiple disease gene queries and a network including both direct and indirect neighbors. This illustrates the ability of mouseNET to identify novel candidates of disease genes based on its multiple-query feature, which cannot be achieved by existing databases nor can be readily extracted from any single genome-scale dataset.

## Discussion

In this study, we combined diverse genetic and genomic data using a probabilistic framework to generate a functional network for the laboratory mouse. Our network accurately predicts functional linkages between mouse genes and covers a broad range of biological processes. We expect this view of the mouse proteome will be an invaluable resource in identifying novel pathway components and understanding system-level organization.

We have demonstrated several applications of our network in this study. First, we characterized the topology of the network and demonstrated that local network topology correlates with biological functions. Also, we used this genomewide view of functional linkages to investigate the relationship between diverse phenotypes and the local configuration of subnetworks. Finally, although network comparison across several species is limited by the sparsity of our current knowledge of physical interactions [40], generation of a functional network based on diverse data types also allowed us to examine the conservation of subnetworks on a global system level.

We provide a searchable interface for the exploration of the mouse functional network (http://mouseNET.princeton.edu). The interface also presents a full analysis of the functional enrichment of networks surrounding the genes(s) of interest and the disease genes in the local network. Through our interface, users could identify the original evidence supporting for specific functional linkages. The website includes integration results generated for the purpose of topological studies (controlled for investigational biases) and of cross-species network alignment studies (by excluding homology data) (http://mouseNET.princeton.edu/supplement/supplemental_data.htm). In the future, new publicly available genome-scale data will be added to our system, which will provide up-to-date support for hypothesis generation for questions ranging from individual protein function prediction to characterization of diverse system-level features.

In this study, we focused on the generation of a global functional network of mouse and demonstrated its wide applicability. Availability of tissue-specific datasets should allow us to generate tissue, cell, and developmental stage-specific network predictions using similar probabilistic frameworks. These tissue or developmental stage-specific networks will be more targeted and will be invaluable to the researchers of individual fields of study.

## Materials and Methods

### Functional Genomic Data Retrieval and Preprocessing

To build a functional network of proteins, we have collected a diverse set of evidence from several databases (Table 1). In order to predict pair-wise protein–protein relationships, all data were preprocessed, as described below, into pair-wise scores, reflecting the similarity between protein pairs. The databases included in our analysis are:

1. Physical interaction data from the Biomolecular Interaction Network Database (BIND) [15], the Database of Interacting Proteins (DIP) [17] and the General Repository for Interaction Datasets (GRID) [16]. We also mapped the interactions in the Online Predicted Human Interaction Database (OPHID) [24] to mouse orthologs via InParanoid [23]. In this process, members of the interactions that have more than one ortholog in mouse were mapped for each of their orthologs. Because physical interaction data are pair-wise and binary (representing the presence or absence of evidence for a physical interaction between a pair of proteins), these datasets were in the format of pair-wise binary scores and were ready to be input into the Bayesian network.
2. Phenotype and disease data from MGI [18] and the Online Mendelian Inheritance in Man (OMIM). The disease association data were mapped to mouse using InParanoid [23]. Based on independence analysis (see below), we found that different phenotypes are highly conditionally dependent on each other, and that the phenotype data and disease data are dependent on each other as well. Thus treating phenotype and disease data as separate evidence nodes in a naïve Bayesian network would cause significant over-estimation of functional relationships between gene pairs that affect the same multiple phenotypes/diseases. As a result, phenotype and disease data were treated as a single evidence node in our Bayesian network, of which the score for the protein pair *j*,*k* will be:
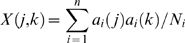
(1)Where *a_i_*(*j*) = 1 if protein *j* has phenotype *i* and *a_i_*(*j*) = 0 otherwise, and *N_i_* is the number of proteins involved in this phenotype/disease; *n* is the total number of phenotypes and diseases. In this way, co-occurrence of rare phenotypes or diseases will be given more weight than common ones. Such calculation allows the transformation from original phenotype/disease profiles to pair-wise scores that reflect the similarity level between a pair of proteins.
3. Homologous functional relationship predictions in yeast from the bioPIXIE system. bioPIXIE is a previously established genomewide prediction of *S. cerevisiae* functional network, which is based on integration of diverse yeast genome-scale datasets [2]. This integrated dataset was used as an input in our mouse interactome by mapping orthologous genes between *S. cerevisiae* and laboratory mouse using InParanoid [23]. The average was taken in the case that orthology mapping results in multiple mapped pair-wise scores in yeast for a single pair in mouse.
4. Expression and Tissue localization datasets from Su et al., 2004, Zhang et al., 2004, and the SAGE database [19]. We chose these three datasets because they represent expression profiles of a wide range of tissue and developmental stages. In total, they included 333 conditions. To make the data suitable as an input to our Bayesian network, we applied the Pearson correlation coefficient *ρ*, to assess levels of co-expression between pairs of genes:
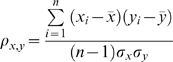
(2)Where *x* and *y* are expression level data vectors of length *n* for two genes, *x̅* and *y̅* are means, and *σ_x_* and *σ_y_* are standard deviations. The correlation coefficients were further Fisher *z*-transfored to ensure comparable, normal distribution [52].

### Filtering Redundant Datasets

In the following section, we applied a naïve Bayes network to integrate all data sources and to predict pair-wise functional relationships. However, the application of a naïve Bayesian framework requires a non-trivial assumption of independence between individual evidence sources, which correspond to different evidence nodes in the naïve Bayes network. To address this issue, we evaluated the conditional independence between datasets and those with significant dependence were merged into a single evidence node. To determine whether two datasets should be merged, we calculated the likelihood ratio of each combination of datasets with and without the assumption of independence.
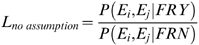
(3)

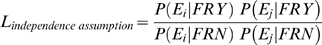
(4)where *E* is the score of the protein pair in dataset *i or j*, a FRY means a positive functional relationship (FR = 1) in gold standard, and FRN means a negative functional relationship (FR = 0).

Two conditionally independent datasets will have similar likelihood ratios calculated by the above two approaches (Figure S6A). In contrast, highly dependent datasets tend to have erroneously high likelihood ratios (Figure S6B) when they are treated as independent ones. After a complete analysis of the independence properties between every dataset pair, we found that phenotype data from MGI and disease data from OMIM are highly dependent on each other. As a result, we treated these phenotype and disease data as a single evidence node in the Bayesian network, and each of the remaining datasets as an individual evidence node.

### Bayesian Network Construction

As data sources are different in their accuracy of measurement as well as relevance for predicting protein functions, creating an accurate network for functional linkages requires a systematic approach that weights and integrates information from individual datasets. We applied a Bayesian network to integrate diverse data and make the final functional linkage predictions (Figure 1A). Specifically, we computed the posterior probability of a functional relationship given all available evidence as follows:
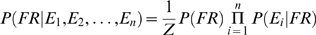
(5)where *FR* represents functional relationship, *E_i_* represents the score of the pair in each dataset *i* and *Z* is a normalization factor. Intuitively, this probability *FR_ij_* for two proteins *i* and *j* represents how likely it is, given existing data and accuracy and coverage of each input dataset, that proteins *i* and *j* participate in the same biological process.

To learn the parameters in this Bayesian framework, we established a gold standard that approximates a true set of functionally related proteins. Mouse Genome Informatics (MGI) maintains curated annotations of Gene Ontology (GO) for mouse [53]. The sources of these annotations include (1) hand annotation from primary literature, (2) electronic annotation based on gene name and symbols, (3) annotation from SwissProt keywords, (4) Enzyme Commision (EC) numbers. These annotation sources are reasonably accurate for our analysis. We defined positive as pairs of proteins that are co-annotated to a specific Biological Process GO term (less than two hundred genes annotated to this GO term) and negatives as those in which both members of the pair have specific annotations but do not share any of them.

To model the posterior distribution given a set of data, we grouped the pair-wise values from each dataset into discrete groups. For binary datasets, for example, physical interactions, it is easy to separate the two categories where 0 means that there is no interaction between the pair, and 1 means that the interaction exists. Continuous pair-wise scores (e.g., expression profiles and phenotype/disease data) require a binning approach for discretization. We observed that for each dataset, the posteriors generally decreases with small fluctuation as the pair-wise score decreases (Figure S7). Thus, to avoid over-fitting to noise in the datasets, discretization was done so as to force the posteriors of the discretized bins to decrease as the average pair-wise score of those bins decreases.

### Network-Based Pathway Component Prediction

An important application of such a functional network is to predict novel pathway components. We therefore applied our network to predict pathway components in KEGG [28]. For a specific pathway, during each iteration, 10 known genes were seeded into the weighted network and the rest of the genes were treated as unknowns. Thus for every other gene, we compute an adjacency to the 10 seeds. This process was repeated three hundred times with random samplings of the seed set. We then calculated the average adjacency for each gene:
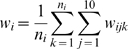
(6)where *w_i_* represents the weight of each gene and *j* represents the seed genes, and *w_ijk_* represents the confidence, as estimated by our integration, of the functional relationship between protein *i* and *j* in iteration *k*. *n_i_* is the number of times gene *i* was not one of the seed genes. The top components and recovery curves were generated based on the ranking of *w_i_*.

### Topological Characterization of the Functional Interactome

To characterize the topology of the functional network, we calculated the connectivity and clustering coefficient *C* of all proteins. The clustering coefficient of a protein gives the probability that its neighbors are connected to each other. In a densely connected module or clique, *C* is close to one. *C* for each of the proteins was calculated as follows [54]:
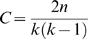
(7)where *n* denotes the number of links between *k* direct interactors.

### Functional Enrichment

We obtained GO annotations [27] from the Mouse Genome Informatics (MGI) [18] on Jan 18, 2007. The enrichment of each GO term was found using a hypergeometric distribution. The most enriched GO terms were represented by the lowest Bonferroni-corrected *p* value [55].

### Implementation, Publicly Available Interface, and Network-Based Gene Function Predictions

To facilitate wide access to the integrated functional network by the biology community, we implemented a web interface (http://mouseNET.princeton.edu) that allows the users to browse our predictions based on single or multiple protein queries. We have implemented a probabilistic algorithm that searches the direct or indirect neighbors with the largest adjacency to the query set [2]. GO term enrichment was calculated for the top neighbors, which facilitates fast discovery of unknown gene function.

We also provide the community with a list of gene function predictions based on our network for proteins with no currently known function. Specifically, we calculated the GO term enrichment of the top 40 nearest neighbors of each gene using the hypergeometric distribution. Then the per-function enrichment of each gene's top neighbors is reported as a Bonferroni-corrected *p*-value and thus their putative function is deduced.

### Experimental Verification

The *Nanog* controllable embryonic stem cell lines were set up and tested by Natalia Ivanova, and were cultured as described [56]. The feeder cells, primary mouse embryonic fibroblasts, were removed before use. To down-regulate *Nanog*, we withdrew the doxycycline (1 g ml^−1^) from the media, but still supplied the cells with all the routine ES cell nutrients (DMEM with 15% FBS (Hyclone), 100 mM MEM non-essential amino acids, 0.1 mM 2-mercaptoethanol, 1 mM l-glutamine (Invitrogen), and 103 U ml-1 of LIF (Chemicon). For the nuclear protein measurement, nuclear protein samples were prepared with nuclear/cytosol fractionation kit (BioVision, catalog number: K266-100). The samples from four different time points were labeled by different isotope (iTRAQ) and then analyzed at a single run of mass spectrometry. We used ProQUANT (Applied Biosystems) and the ProGROUP (Applied Biosystems) software to identify proteins. The experiment was repeated three times. Proteins detected more than twice were included in the analysis and the average values were used.

## Supporting Information

Dataset S1 Functional composition and biases of each data source and the integrated result.
(0.41 MB XLS)

Dataset S2 Expert curation for a selected set of gene function predictions based on the network.
(0.07 MB XLS)

Dataset S3 Functional biases of conserved and non-conserved sub-network.
(0.09 MB XLS)

Figure S1 The functional composition of the integrated results and individual datasets.
(0.14 MB TIF)

Figure S2 Performance of the integrated interactome in predicting the components of six major pathways in development.
(1.57 MB TIF)

Figure S3 Connectivity (at 0.3 cutoff in confidence) versus clustering coefficient.
(0.43 MB TIF)

Figure S4 Connectivity and phenotypic effects in networks integrated using both individual experimental evidence and large-scale genomic data.
(0.67 MB TIF)

Figure S5 Illustration of the mouseNET interface.
(1.20 MB TIF)

Figure S6 Example of a conditionally independent pair of datasets and a conditionally dependent dataset pair.
(0.24 MB TIF)

Figure S7 The general trend of posteriors for continuous datasets.
(0.13 MB TIF)

Figure S8 The distribution of Log2 changes in protein expression level on the fifth day after Nanog knock-down for 1148 proteins detected in the nucleus.
(0.11 MB TIF)

Table S1 Mapping of phenotypes and MP index in MGI.
(0.05 MB DOC)

Table S2 Literature evidence for novel components (not currently annotated to MAPK in KEGG or GO) predicted to be involved in MAPK pathway.
(0.09 MB DOC)

Text S1 Supplementary figure and tables.
(5.07 MB DOC)
