# Supplementary figures and images for: A Genomewide Functional Network for the Laboratory Mouse
Source: PLoS Comput Biol. 2008 Sep 26;4(9):e1000165. doi: 10.1371/journal.pcbi.1000165 (PMC2527685; doi:10.1371/journal.pcbi.1000165)

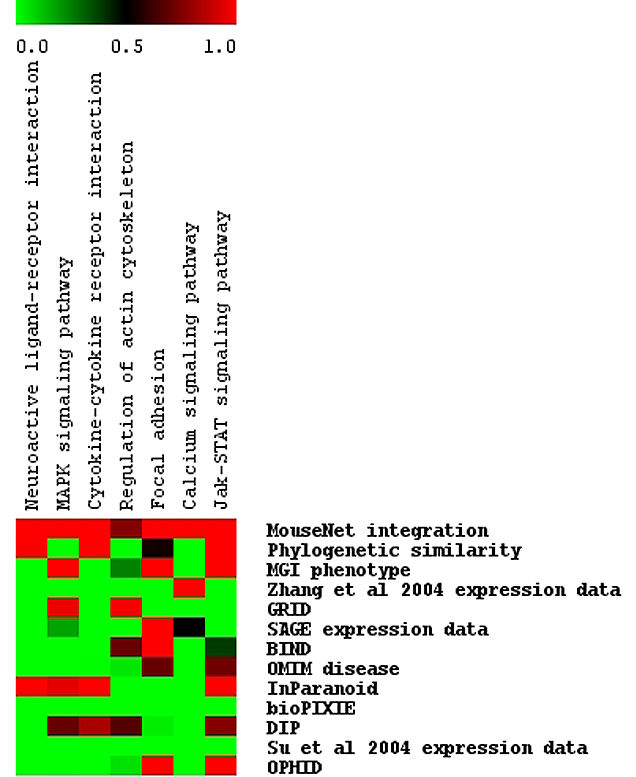

Supplement: Figure S1 — The functional composition of the integrated results and individual datasets. (0.14 MB TIF) [file pcbi.1000165.s004.tif]

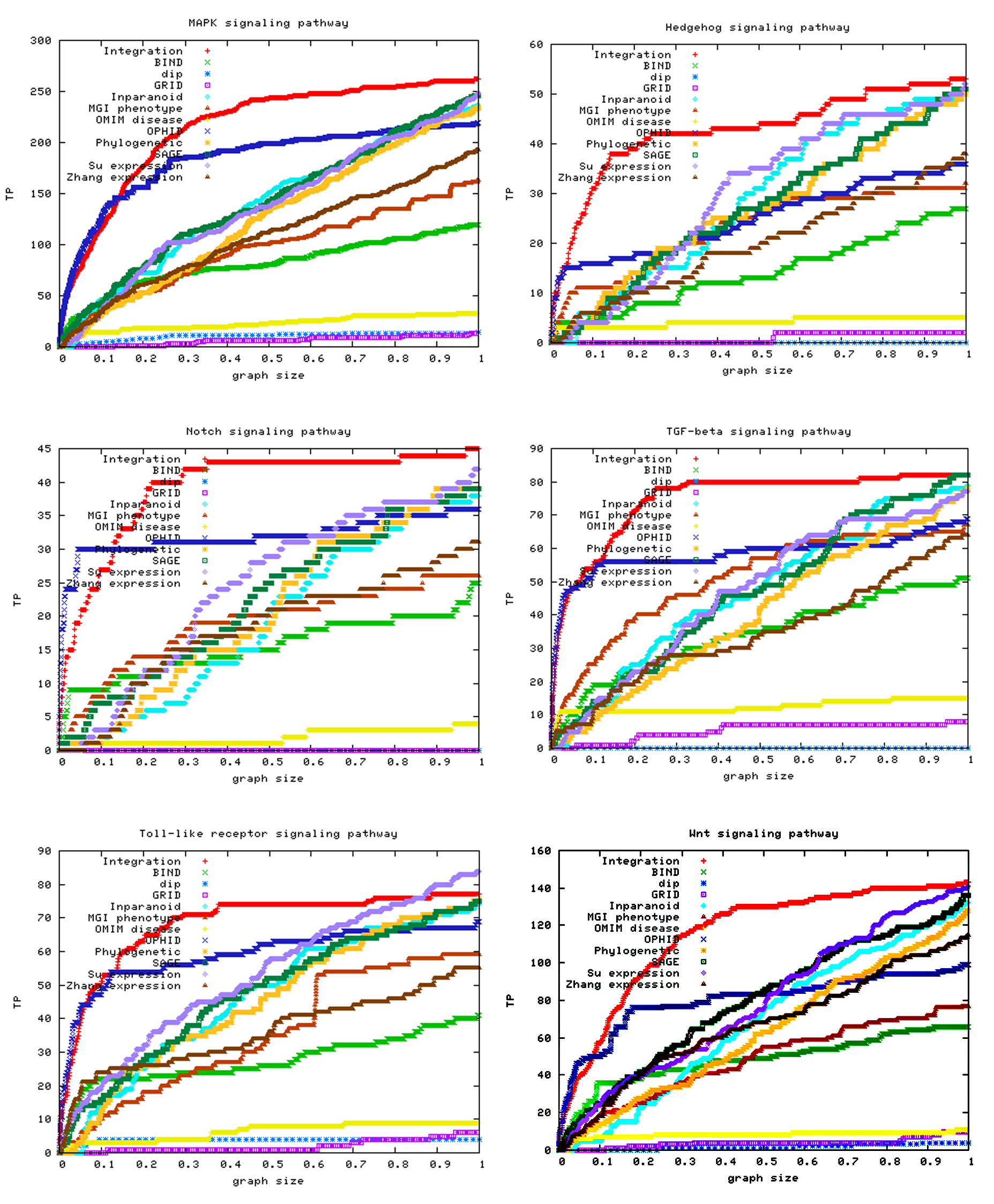

Supplement: Figure S2 — Performance of the integrated interactome in predicting the components of six major pathways in development. (1.57 MB TIF) [file pcbi.1000165.s005.tif]

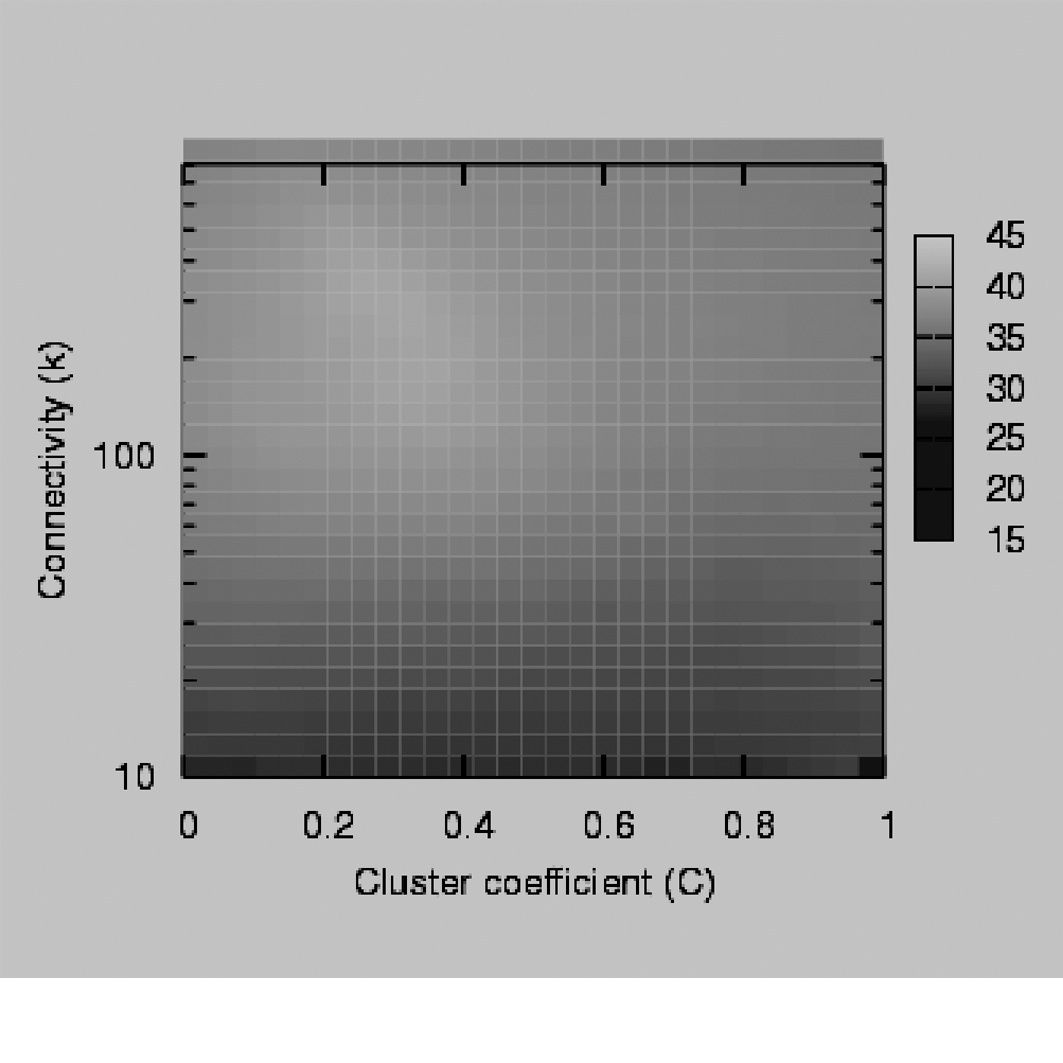

Supplement: Figure S3 — Connectivity (at 0.3 cutoff in confidence) versus clustering coefficient. (0.43 MB TIF) [file pcbi.1000165.s006.tif]

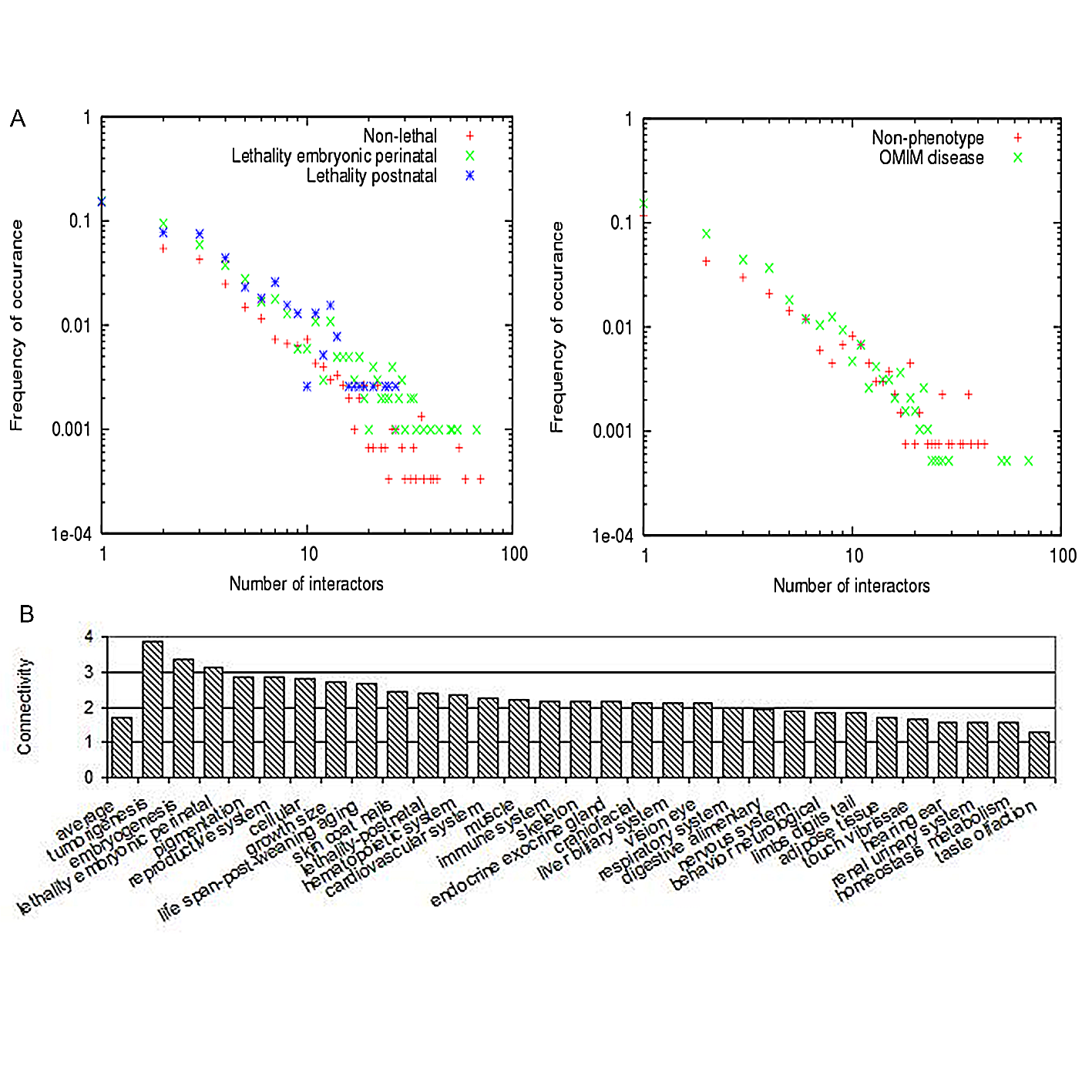

Supplement: Figure S4 — Connectivity and phenotypic effects in networks integrated using both individual experimental evidence and large-scale genomic data. (0.67 MB TIF) [file pcbi.1000165.s007.tif]

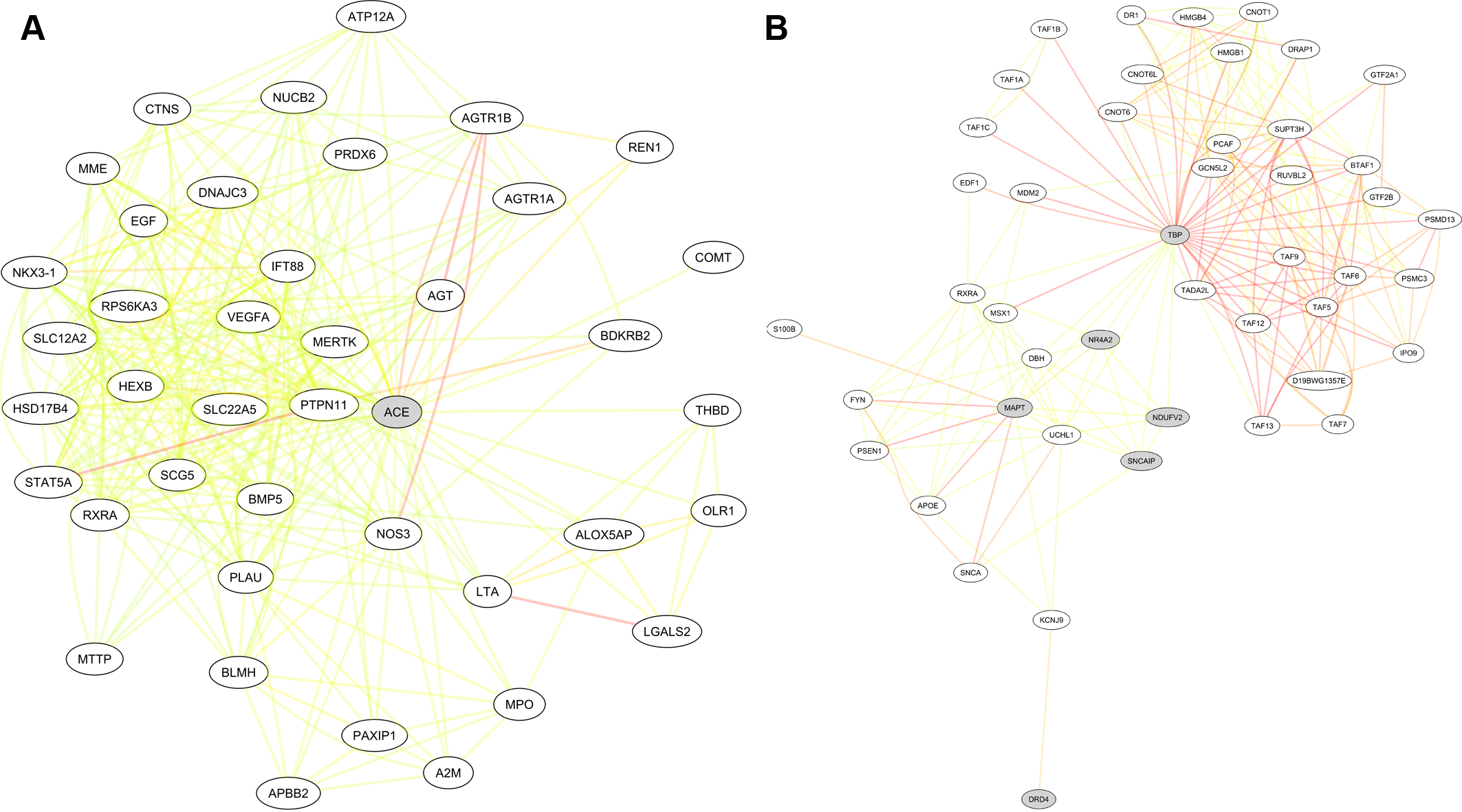

Supplement: Figure S5 — Illustration of the mouseNET interface. (1.20 MB TIF) [file pcbi.1000165.s008.tif]

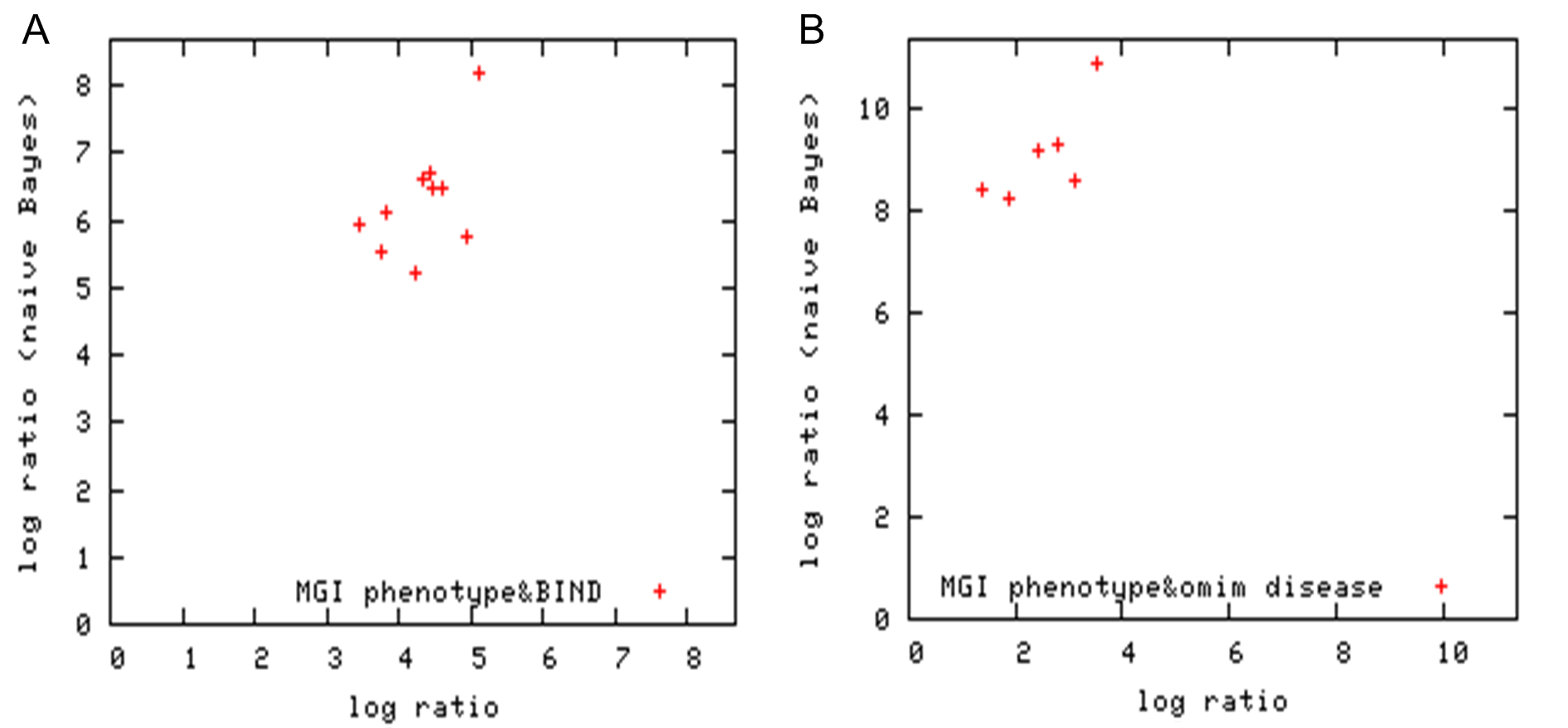

Supplement: Figure S6 — Example of a conditionally independent pair of datasets and a conditionally dependent dataset pair. (0.24 MB TIF) [file pcbi.1000165.s009.tif]

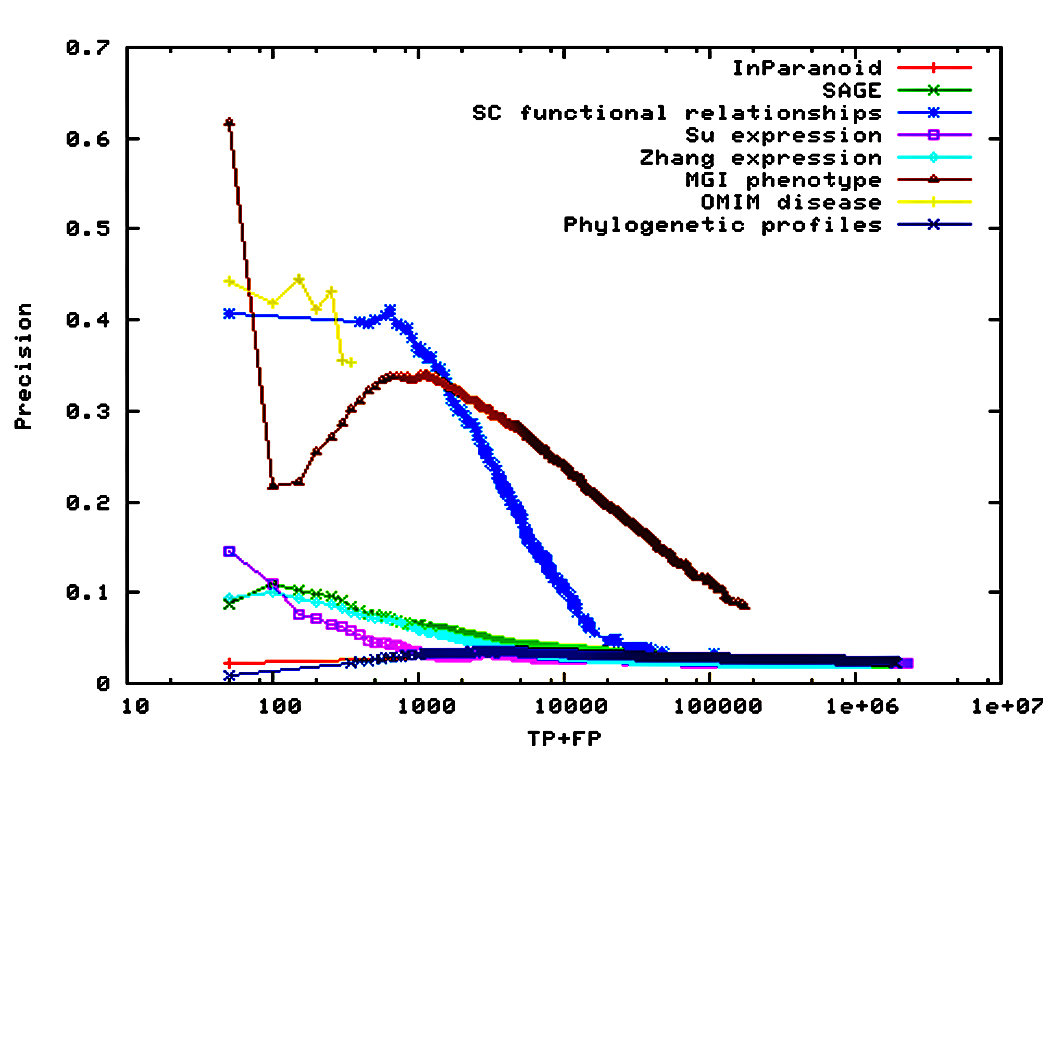

Supplement: Figure S7 — The general trend of posteriors for continuous datasets. (0.13 MB TIF) [file pcbi.1000165.s010.tif]

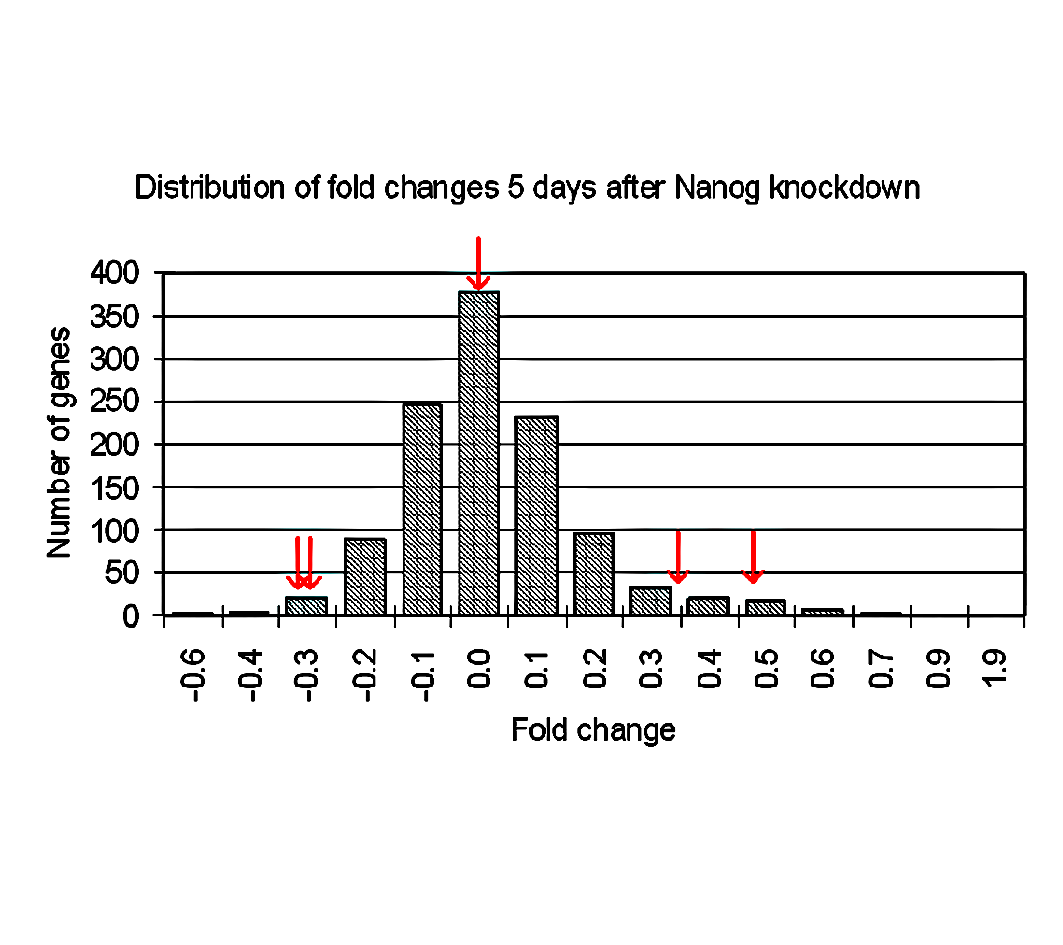

Supplement: Figure S8 — The distribution of Log2 changes in protein expression level on the fifth day after Nanog knock-down for 1148 proteins detected in the nucleus. (0.11 MB TIF) [file pcbi.1000165.s011.tif]
