# Supplementary material for: A Genomewide Functional Network for the Laboratory Mouse
Source: PLoS Comput Biol. 2008 Sep 26;4(9):e1000165. doi: 10.1371/journal.pcbi.1000165 (PMC2527685; doi:10.1371/journal.pcbi.1000165)
Supplement: Table S1 — Mapping of phenotypes and MP index in MGI. (0.05 MB DOC) [file pcbi.1000165.s012.doc]

**Table S1. Mapping of phenotypes and MP index in MGI.**

| MP number | Phenotype description |
| --- | --- |
| [MP_0001186](ftp://ftp.informatics.jax.org/pub/gbrowse/Jan2007/MP_0001186_pigmentation.gff) | Pigmentation |
| [MP_0002006](ftp://ftp.informatics.jax.org/pub/gbrowse/Jan2007/MP_0002006_tumorigenesis.gff) | Tumorigenesis |
| [MP_0003631](ftp://ftp.informatics.jax.org/pub/gbrowse/Jan2007/MP_0003631_nervous_system.gff) | Nervous system |
| [MP_0005367](ftp://ftp.informatics.jax.org/pub/gbrowse/Jan2007/MP_0005367_renal_urinary_system.gff) | Renal urinary system |
| [MP_0005369](ftp://ftp.informatics.jax.org/pub/gbrowse/Jan2007/MP_0005369_muscle.gff) | Muscle |
| [MP_0005370](ftp://ftp.informatics.jax.org/pub/gbrowse/Jan2007/MP_0005370_liver_biliary_system.gff) | Liver biliary system |
| [MP_0005371](ftp://ftp.informatics.jax.org/pub/gbrowse/Jan2007/MP_0005371_limbs_digits_tail.gff) | Limbs digits tail |
| [MP_0005372](ftp://ftp.informatics.jax.org/pub/gbrowse/Jan2007/MP_0005372_life_span-post-weaning_aging.gff) | Life span-post-weaning aging |
| [MP_0005373](ftp://ftp.informatics.jax.org/pub/gbrowse/Jan2007/MP_0005373_lethality-postnatal.gff) | Lethality-postnatal |
| [MP_0005374](ftp://ftp.informatics.jax.org/pub/gbrowse/Jan2007/MP_0005374_lethality-embryonic_perinatal.gff) | Lethality-embyonic perinatal |
| [MP_0005375](ftp://ftp.informatics.jax.org/pub/gbrowse/Jan2007/MP_0005375_adipose_tissue.gff) | Adipose tissue |
| [MP_0005376](ftp://ftp.informatics.jax.org/pub/gbrowse/Jan2007/MP_0005376_homeostasis_metabolism.gff) | Homeostasis metabolism |
| [MP_0005377](ftp://ftp.informatics.jax.org/pub/gbrowse/Jan2007/MP_0005377_hearing_ear.gff) | Hearing ear |
| [MP_0005378](ftp://ftp.informatics.jax.org/pub/gbrowse/Jan2007/MP_0005378_growth_size.gff) | Growth size |
| [MP_0005379](ftp://ftp.informatics.jax.org/pub/gbrowse/Jan2007/MP_0005379_endocrine_exocrine_gland.gff) | Endocrine exocrine gland |
| [MP_0005380](ftp://ftp.informatics.jax.org/pub/gbrowse/Jan2007/MP_0005380_embryogenesis.gff) | Embryogenesis |
| [MP_0005381](ftp://ftp.informatics.jax.org/pub/gbrowse/Jan2007/MP_0005381_digestive_alimentary.gff) | Digestive alimentary |
| [MP_0005382](ftp://ftp.informatics.jax.org/pub/gbrowse/Jan2007/MP_0005382_craniofacial.gff) | Craniofacial |
| [MP_0005384](ftp://ftp.informatics.jax.org/pub/gbrowse/Jan2007/MP_0005384_cellular.gff) | Cellular |
| [MP_0005385](ftp://ftp.informatics.jax.org/pub/gbrowse/Jan2007/MP_0005385_cardiovascular_system.gff) | Cardiovascular system |
| [MP_0005386](ftp://ftp.informatics.jax.org/pub/gbrowse/Jan2007/MP_0005386_behavior_neurological.gff) | Behavior neurological |
| [MP_0005387](ftp://ftp.informatics.jax.org/pub/gbrowse/Jan2007/MP_0005387_immune_system.gff) | Immune system |
| [MP_0005388](ftp://ftp.informatics.jax.org/pub/gbrowse/Jan2007/MP_0005388_respiratory_system.gff) | Respiratory system |
| [MP_0005389](ftp://ftp.informatics.jax.org/pub/gbrowse/Jan2007/MP_0005389_reproductive_system.gff) | Reproductive system |
| [MP_0005390](ftp://ftp.informatics.jax.org/pub/gbrowse/Jan2007/MP_0005390_skeleton.gff) | Skeleton |
| [MP_0005391](ftp://ftp.informatics.jax.org/pub/gbrowse/Jan2007/MP_0005391_vision_eye.gff) | Vision eye |
| [MP_0005392](ftp://ftp.informatics.jax.org/pub/gbrowse/Jan2007/MP_0005392_touch_vibrissae.gff) | Touch vibrissae |
| MP_0005397 | Hematopoietic system |
| [MP_0005393](ftp://ftp.informatics.jax.org/pub/gbrowse/Jan2007/MP_0005393_skin_coat_nails.gff) | Skin coat nails |
| [MP_0005394](ftp://ftp.informatics.jax.org/pub/gbrowse/Jan2007/MP_0005394_taste_olfaction.gff) | Taste olfaction |
