# Supplementary material for: A Genomewide Functional Network for the Laboratory Mouse
Source: PLoS Comput Biol. 2008 Sep 26;4(9):e1000165. doi: 10.1371/journal.pcbi.1000165 (PMC2527685; doi:10.1371/journal.pcbi.1000165)
Supplement: Table S2 — Literature evidence for novel components (not currently annotated to MAPK in KEGG or GO) predicted to be involved in MAPK pathway. (0.09 MB DOC) [file pcbi.1000165.s013.doc]

**Table S2. Literature evidence for novel components (not currently annotated to MAPK in KEGG or GO) predicted to be involved in MAPK pathway.**

| 1 | MGI:88373 | Cebpb | 1-4 | p38 MAPKs regulate TEF-1 and C/EBPbeta transcriptional activity in the absence of environmental stress (mouse)  Sequential phosphorylation of CCAAT enhancer-binding protein beta by MAPK and glycogen synthase kinase 3beta is required for adipogenesis (mouse).  transcription factor C/EBPbeta and the MAPK pathway play key roles in the response of the plasminogen gene to IL-6 (mouse)  IGFBP-1 may support liver regeneration at least in part via its effect on MAPK/ERK and C/EBP beta activities (mouse) |
| --- | --- | --- | --- | --- |
| 2 | MGI:1338938 | Bmpr1A | N/A |  |
| 3 | MGI:101900 | Mmp14 | 5 | MT1-MMP activity is regulated by ERK 1/2- and p38 MAPK-modulated TIMP-2 expression which controls TGF-beta1-induced pericellular collagenolysis (human) |
| 4 | MGI:1277166 | Sp3 | N/A |  |
| 5 | MGI:96677 | Kit (oncogene) | GO  ^6^ | IGI: positive regulation of MAPK activity (mouse) |
| 6 | MGI:98297 | Shh | N/A |  |
| 7 | MGI:96969 | Met (proto-oncogene) | GO  ^7^ | IPI: activation of MAPK activity (mouse) |
| 8 | MGI:88071 | Arnt | N/A |  |
| 9 | MGI:97350 | Nkx2-5 | N/A |  |
| 10 | MGI:97747 | Pparg | N/A |  |
| 11 | MGI:97805 | Ptpn1 | N/A |  |
| 12 | MGI:95602 | Fyn | 8,9 | the dominance of the Fyn/p38 MAPK pathway in driving IL-4 production (mouse)  Fyn stimulates the ERK/MAPK pathway in primary T cells but has little influence on the mobilization of Ca2+. Our result suggests that Fyn activates ERK via a different upstream signaling route. (mouse) |
| 13 | MGI:97874 | Rb1 | 10-13 | These studies highlight p38 MAPK, HBP1, and RB as important components for a premature-senescence pathway with possible clinical relevance to breast cancer. (human)  RB1 is phosphorylated by MAPK1. This interaction was modeled on a demonstrated interaction between human KIAA1536 and MAPK1 from an unspecified species. (human)  Interact with MAPK9 (human)  retinoblastoma protein (pRb) plays a pivotal role in adipogenesis by suppressing MAPK activity (mouse) |
| 14 | MGI:97610 | Plat | 14,15 | TNF-alpha impairs fibrinolytic capacity in vascular endothelial cells by a NF-kappaB and p38 MAPK-dependent suppression of t-PA (human)  Interact with MAPK1 (human)  Interact with MAPK3 (human) |
| 15 | MGI:88180 | Bmp4 | N/A |  |
| 16 | MGI:1098280 | Crebbp | 16 | activation of CREB-binding protein and inhibition of MAPK has a role in cAMP-dependent protein kinase type I regulation of ethanol-induced cAMP response element-mediated gene expression (human) |
| 17 | MGI:97603 | Pkd1 | N/A |  |
| 18 | MGI:97323 | Ngfr | 17 | Data show that sustained activation of p38(MAPK) is essential for the death cascade following exposure of Ewing's sarcoma tumor cells to bFGF and provide evidence that activation of p38(MAPK) results in an up-regulation of the death receptor p75(NTR) (human). |
| 19 | MGI:88388 | Cftr | 18 | A single allelic CFTR mutation is sufficient to augment IL-8 secretion in response to LPS. This is not a result of increased LPS receptor expression but, rather, is associated with alterations in MAPK signaling (human). |
| 20 | MGI:104311 | Ptger4 | 19 | We concluded that PGE2 stimulates the BNP promoter mainly via EP4, PKA, Rap, and p42/44 MAPK (human) |
| 21 | MGI:1100846 | Pparbp | N/A |  |
| 22 | MGI:96748 | Lamp2 | N/A |  |
| 23 | MGI:105923 | Ednra | N/A |  |
| 24 | MGI:98214 | Rxra | N/A |  |
| 25 | MGI:97603 | Pkd1 | N/A |  |
| 26 | MGI:1859631 | Pdgfc | N/A |  |
