# Supplementary material for: A Genomewide Functional Network for the Laboratory Mouse
Source: PLoS Comput Biol. 2008 Sep 26;4(9):e1000165. doi: 10.1371/journal.pcbi.1000165 (PMC2527685; doi:10.1371/journal.pcbi.1000165)
Supplement: Text S1 — Supplementary figure and tables. (5.07 MB DOC) [file pcbi.1000165.s014.doc]

**Supplementary figures and tables for**

**A genome-wide functional network for the laboratory mouse**

Yuanfang Guan1,2, Chad L. Myers3, Rong Lu2, Ihor R. Lemischka2, Carol J. Bult4, Olga G. Troyanskaya1,3*,

1Lewis-Sigler Institute for Integrative Genomics, Carl Icahn Laboratory, 2Department of Molecular Biology, 3­­Department of Computer Science, Princeton University, Princeton, NJ 08544, 4The Jackson Laboratory 600 Main Street, Bar Harbor, Maine, USA.

*****Correspondence: ogt@cs.princeton.edu

**Figure S1. The functional composition of the integrated results and individual datasets.** The graph represents the enrichment (in cumulative hypergeometric distribution function) of each set of genes in the seven largest KEGG pathways, where higher value indicates better coverage. This result shows the distribution of functions for pairs having above-prior value in the integrated results, binary data (physical interaction) and the functional distribution of the top 10,000 true positive pairs of continuous datasets.


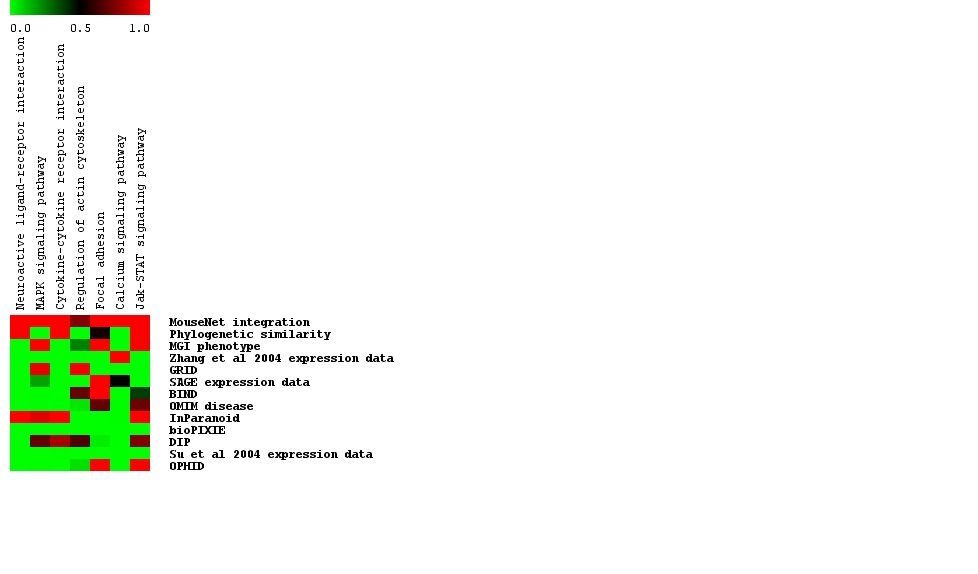


**Figure S2. Performance of the integrated interactome in predicting the components of six major pathways in development.** This figure plots the number of true positive pairs predicted (TP) versus the fraction of the whole network.

**
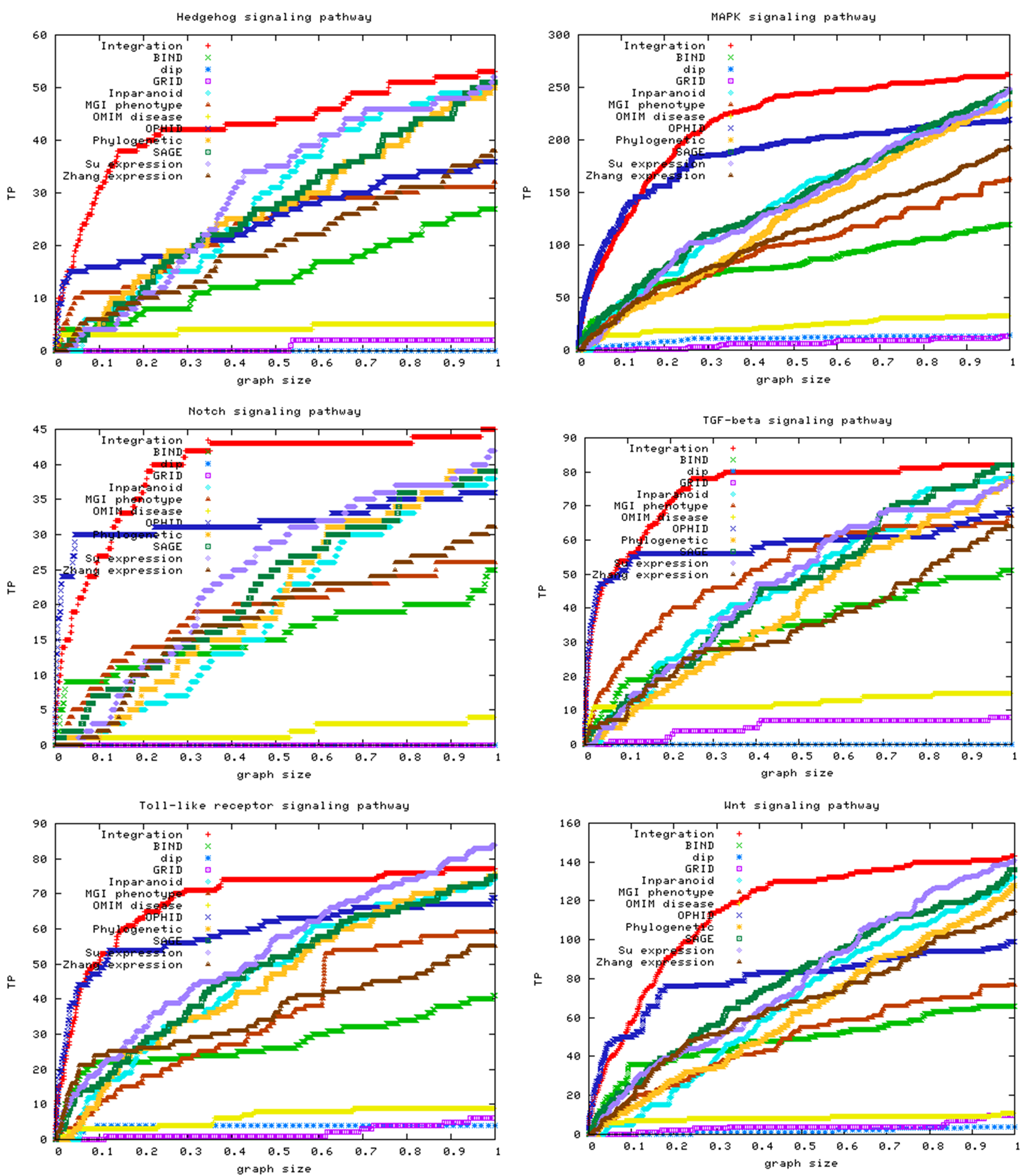
**

**Figure S3. Connectivity (at 0.3 cutoff in confidence) versus clustering coefficient.** The color represents the number of processes presented in that gene’s local network (top 40 neighbors). At the same level of connectivity, proteins with smaller clustering coefficients tend to participate in more pathways. This figure and Figure 5B together shows that the relationship between clustering coefficient and the number of pathways a gene participates in is robust against different cutoffs.

**
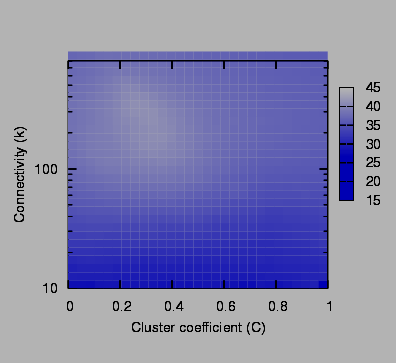
**

**Figure S4**. **Connectivity and phenotypic effects in networks integrated using both individual experimental evidence and large-scale genomic data.** A.Comparison of connectivity between essential and non-essential genes (left), between genes whose orthologous mutant causing a disease and genes causing no phenotype (right). These results were based on network re-integrated by excluding any phenotypic or disease data, but including the datasets involving individual investigations. B.The average number of interactions each gene has and their phenotypic effects (network integrated without phenotypic data).

**
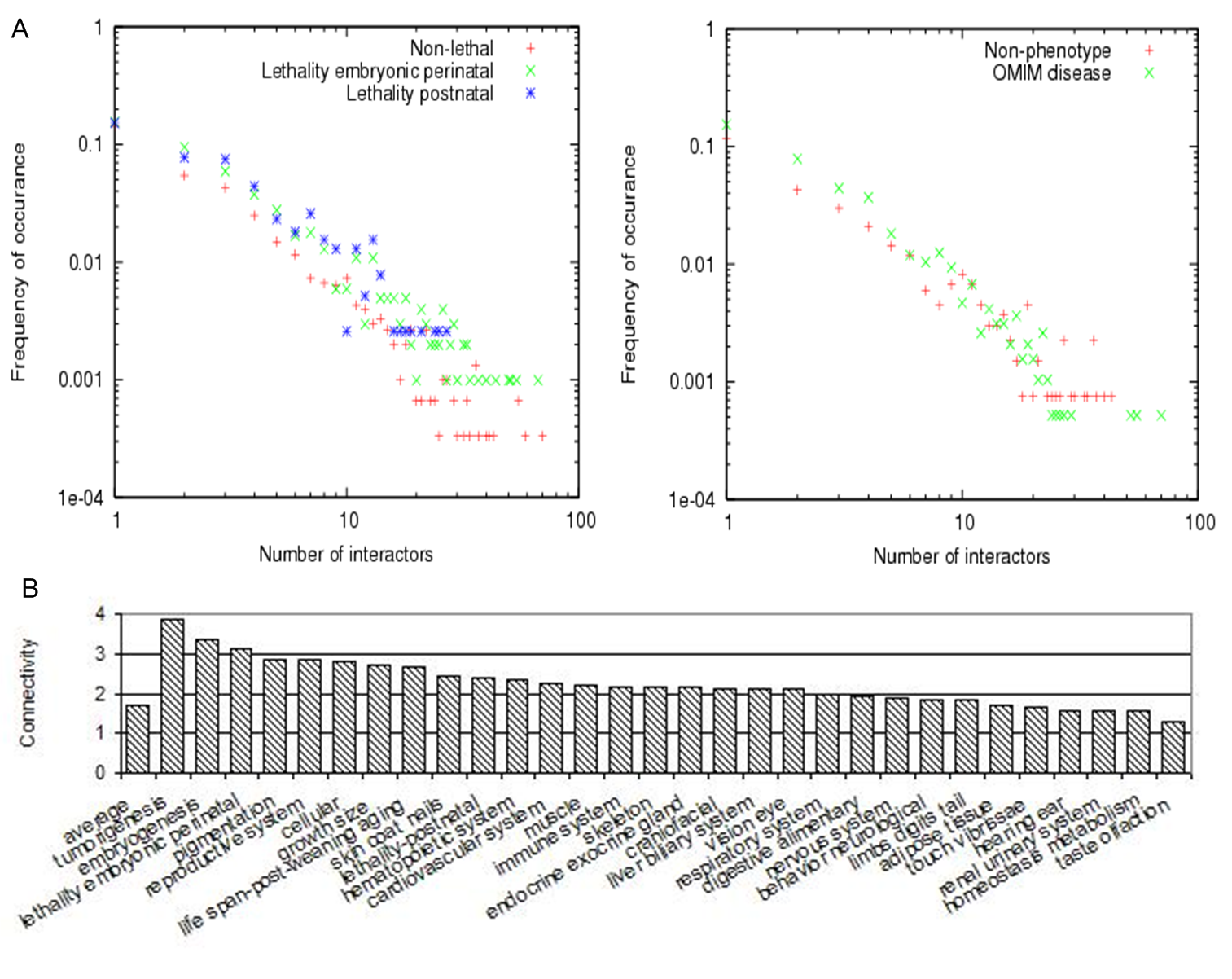
**

**Figure S5. Illustration of the mouseNET interface.** A. MouseNET identifies unrelated processes a gene participating in.The nodes represent proteins (queries in grey), and the edges represent the confidence of functional association between them. The local network of *Ace* predicts that it is involved in two independent processes: blood pressure regulation (GO:0008217) and menstrual cycle phase (GO:0022601). B. MouseNET identifies disease-related genes and discovers novel candidates.Six genes involved in Parkinson’s disease were entered into mouseNET, and the top three genes (*Uchl1*, *Dbh* and *Snca*) returned were also annotated to this disease. The fourth gene *Msx1* is a novel candidate of this disease, evident by its connection with the queries and their direct neighbors.

**
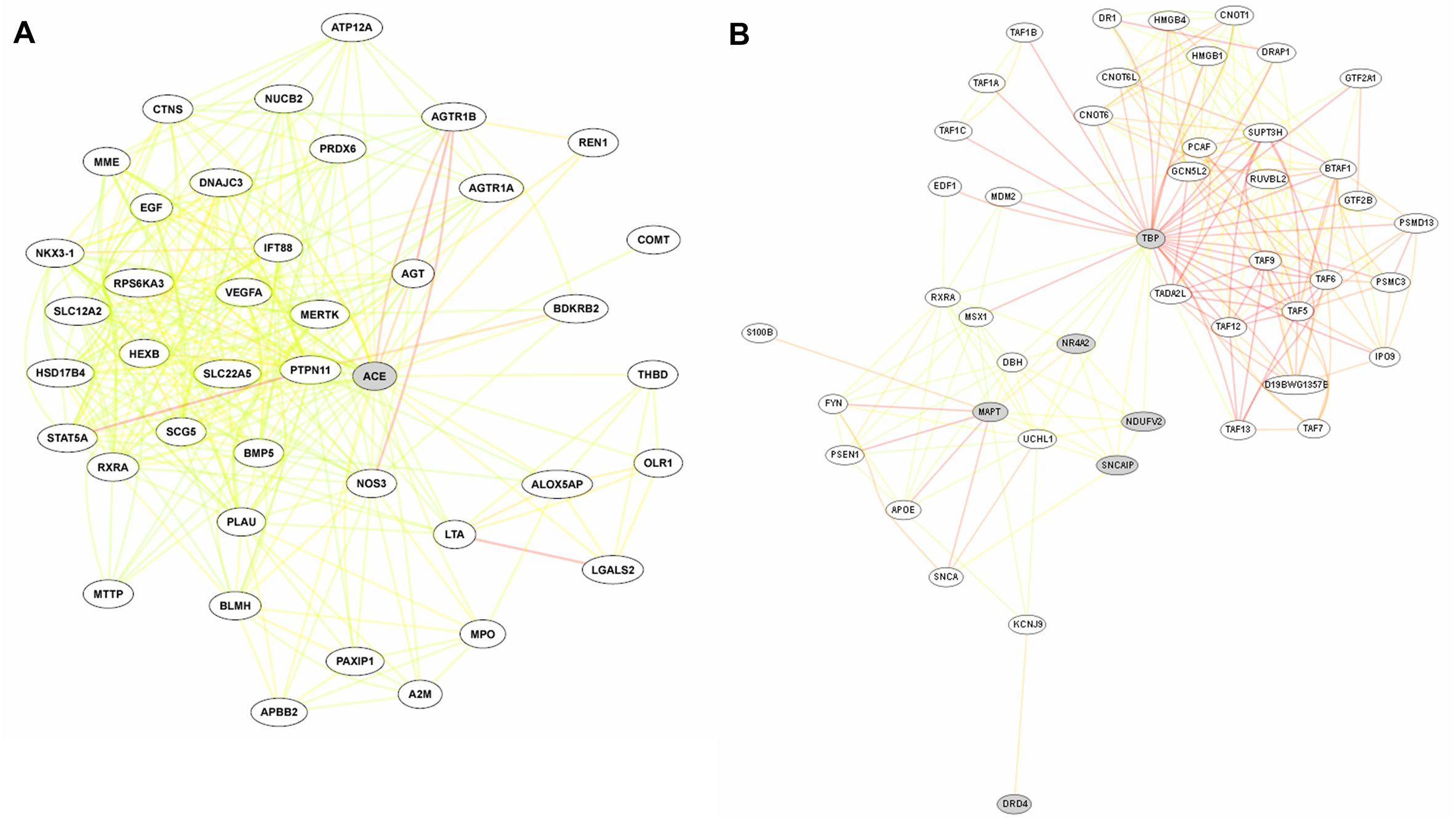
**

**Figure S6.** **Example of a conditionally independent pair of datasets and a conditionally dependent dataset pair.** For each discrete bin combination (combination of the states of the evidence nodes) of the two datasets, the estimated likelihood ratio by naïve Bayes was plotted against the likelihood ratio calculated without an independence assumption. A. Phenotype profile and BIND physical interaction. Likelihood ratios estimated assuming independence and without such an assumption are similar to each other, indicating that the two datasets are conditionally independent of each other. B.Phenotype profiles and OMIM disease data. Likelihood ratios estimated assuming independence are much higher than those estimated without such an assumption, indicating the two datasets are conditionally dependent. Data set pairs as such should therefore be combined into a single evidence node in the Naïve Bayes framework.

**
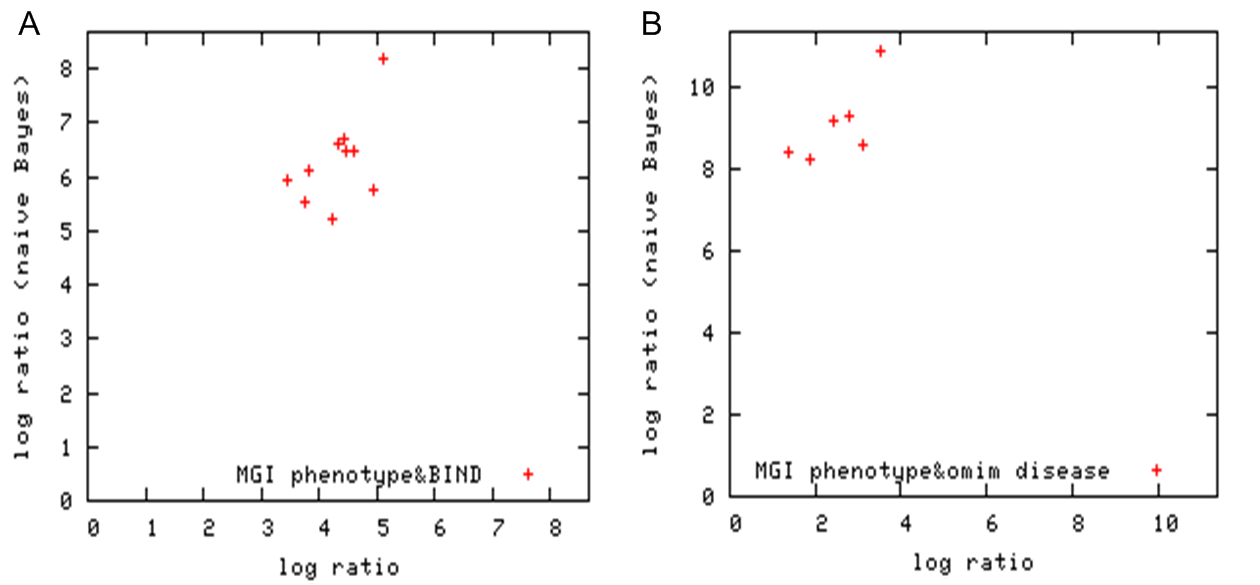
**

**Figure S7. The general trend of posteriors for continuous datasets.** This figure plots the precision versus the number of true positives (TP) +false positives (FP) pairs. We observed that, with some fluctuation, precision generally decreases as the total number of (TP+FP) pairs increase. Therefore, for each continuous dataset, precision in general decreases as the pair-wise scores decrease.

**
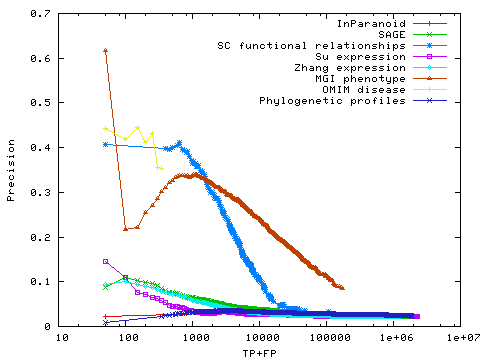
**

**Figure S8. The distribution of Log2 changes in protein expression level on the fifth day after *Nanog* knock-down for 1148 proteins detected in the nucleus.** Arrows point to the values of the top five
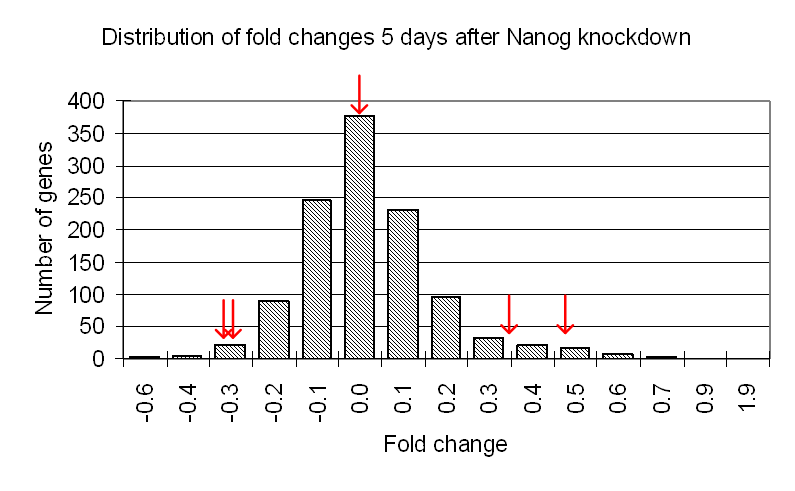
proteins predicted by our functional network and detected in the nucleus.

**Table S1. Mapping of phenotypes and MP index in MGI.**

| MP number | Phenotype description |
| --- | --- |
| [MP_0001186](ftp://ftp.informatics.jax.org/pub/gbrowse/Jan2007/MP_0001186_pigmentation.gff) | Pigmentation |
| [MP_0002006](ftp://ftp.informatics.jax.org/pub/gbrowse/Jan2007/MP_0002006_tumorigenesis.gff) | Tumorigenesis |
| [MP_0003631](ftp://ftp.informatics.jax.org/pub/gbrowse/Jan2007/MP_0003631_nervous_system.gff) | Nervous system |
| [MP_0005367](ftp://ftp.informatics.jax.org/pub/gbrowse/Jan2007/MP_0005367_renal_urinary_system.gff) | Renal urinary system |
| [MP_0005369](ftp://ftp.informatics.jax.org/pub/gbrowse/Jan2007/MP_0005369_muscle.gff) | Muscle |
| [MP_0005370](ftp://ftp.informatics.jax.org/pub/gbrowse/Jan2007/MP_0005370_liver_biliary_system.gff) | Liver biliary system |
| [MP_0005371](ftp://ftp.informatics.jax.org/pub/gbrowse/Jan2007/MP_0005371_limbs_digits_tail.gff) | Limbs digits tail |
| [MP_0005372](ftp://ftp.informatics.jax.org/pub/gbrowse/Jan2007/MP_0005372_life_span-post-weaning_aging.gff) | Life span-post-weaning aging |
| [MP_0005373](ftp://ftp.informatics.jax.org/pub/gbrowse/Jan2007/MP_0005373_lethality-postnatal.gff) | Lethality-postnatal |
| [MP_0005374](ftp://ftp.informatics.jax.org/pub/gbrowse/Jan2007/MP_0005374_lethality-embryonic_perinatal.gff) | Lethality-embyonic perinatal |
| [MP_0005375](ftp://ftp.informatics.jax.org/pub/gbrowse/Jan2007/MP_0005375_adipose_tissue.gff) | Adipose tissue |
| [MP_0005376](ftp://ftp.informatics.jax.org/pub/gbrowse/Jan2007/MP_0005376_homeostasis_metabolism.gff) | Homeostasis metabolism |
| [MP_0005377](ftp://ftp.informatics.jax.org/pub/gbrowse/Jan2007/MP_0005377_hearing_ear.gff) | Hearing ear |
| [MP_0005378](ftp://ftp.informatics.jax.org/pub/gbrowse/Jan2007/MP_0005378_growth_size.gff) | Growth size |
| [MP_0005379](ftp://ftp.informatics.jax.org/pub/gbrowse/Jan2007/MP_0005379_endocrine_exocrine_gland.gff) | Endocrine exocrine gland |
| [MP_0005380](ftp://ftp.informatics.jax.org/pub/gbrowse/Jan2007/MP_0005380_embryogenesis.gff) | Embryogenesis |
| [MP_0005381](ftp://ftp.informatics.jax.org/pub/gbrowse/Jan2007/MP_0005381_digestive_alimentary.gff) | Digestive alimentary |
| [MP_0005382](ftp://ftp.informatics.jax.org/pub/gbrowse/Jan2007/MP_0005382_craniofacial.gff) | Craniofacial |
| [MP_0005384](ftp://ftp.informatics.jax.org/pub/gbrowse/Jan2007/MP_0005384_cellular.gff) | Cellular |
| [MP_0005385](ftp://ftp.informatics.jax.org/pub/gbrowse/Jan2007/MP_0005385_cardiovascular_system.gff) | Cardiovascular system |
| [MP_0005386](ftp://ftp.informatics.jax.org/pub/gbrowse/Jan2007/MP_0005386_behavior_neurological.gff) | Behavior neurological |
| [MP_0005387](ftp://ftp.informatics.jax.org/pub/gbrowse/Jan2007/MP_0005387_immune_system.gff) | Immune system |
| [MP_0005388](ftp://ftp.informatics.jax.org/pub/gbrowse/Jan2007/MP_0005388_respiratory_system.gff) | Respiratory system |
| [MP_0005389](ftp://ftp.informatics.jax.org/pub/gbrowse/Jan2007/MP_0005389_reproductive_system.gff) | Reproductive system |
| [MP_0005390](ftp://ftp.informatics.jax.org/pub/gbrowse/Jan2007/MP_0005390_skeleton.gff) | Skeleton |
| [MP_0005391](ftp://ftp.informatics.jax.org/pub/gbrowse/Jan2007/MP_0005391_vision_eye.gff) | Vision eye |
| [MP_0005392](ftp://ftp.informatics.jax.org/pub/gbrowse/Jan2007/MP_0005392_touch_vibrissae.gff) | Touch vibrissae |
| MP_0005397 | Hematopoietic system |
| [MP_0005393](ftp://ftp.informatics.jax.org/pub/gbrowse/Jan2007/MP_0005393_skin_coat_nails.gff) | Skin coat nails |
| [MP_0005394](ftp://ftp.informatics.jax.org/pub/gbrowse/Jan2007/MP_0005394_taste_olfaction.gff) | Taste olfaction |

**Table S2. Literature evidence for novel components (not currently annotated to MAPK in KEGG or GO) predicted to be involved in MAPK pathway.**

| 1 | MGI:88373 | Cebpb | 1-4 | p38 MAPKs regulate TEF-1 and C/EBPbeta transcriptional activity in the absence of environmental stress (mouse)  Sequential phosphorylation of CCAAT enhancer-binding protein beta by MAPK and glycogen synthase kinase 3beta is required for adipogenesis (mouse).  transcription factor C/EBPbeta and the MAPK pathway play key roles in the response of the plasminogen gene to IL-6 (mouse)  IGFBP-1 may support liver regeneration at least in part via its effect on MAPK/ERK and C/EBP beta activities (mouse) |
| --- | --- | --- | --- | --- |
| 2 | MGI:1338938 | Bmpr1A | N/A |  |
| 3 | MGI:101900 | Mmp14 | 5 | MT1-MMP activity is regulated by ERK 1/2- and p38 MAPK-modulated TIMP-2 expression which controls TGF-beta1-induced pericellular collagenolysis (human) |
| 4 | MGI:1277166 | Sp3 | N/A |  |
| 5 | MGI:96677 | Kit (oncogene) | GO  6 | IGI: positive regulation of MAPK activity (mouse) |
| 6 | MGI:98297 | Shh | N/A |  |
| 7 | MGI:96969 | Met (proto-oncogene) | GO  7 | IPI: activation of MAPK activity (mouse) |
| 8 | MGI:88071 | Arnt | N/A |  |
| 9 | MGI:97350 | Nkx2-5 | N/A |  |
| 10 | MGI:97747 | Pparg | N/A |  |
| 11 | MGI:97805 | Ptpn1 | N/A |  |
| 12 | MGI:95602 | Fyn | 8,9 | the dominance of the Fyn/p38 MAPK pathway in driving IL-4 production (mouse)  Fyn stimulates the ERK/MAPK pathway in primary T cells but has little influence on the mobilization of Ca2+. Our result suggests that Fyn activates ERK via a different upstream signaling route. (mouse) |
| 13 | MGI:97874 | Rb1 | 10-13 | These studies highlight p38 MAPK, HBP1, and RB as important components for a premature-senescence pathway with possible clinical relevance to breast cancer. (human)  RB1 is phosphorylated by MAPK1. This interaction was modeled on a demonstrated interaction between human KIAA1536 and MAPK1 from an unspecified species. (human)  Interact with MAPK9 (human)  retinoblastoma protein (pRb) plays a pivotal role in adipogenesis by suppressing MAPK activity (mouse) |
| 14 | MGI:97610 | Plat | 14,15 | TNF-alpha impairs fibrinolytic capacity in vascular endothelial cells by a NF-kappaB and p38 MAPK-dependent suppression of t-PA (human)  Interact with MAPK1 (human)  Interact with MAPK3 (human) |
| 15 | MGI:88180 | Bmp4 | N/A |  |
| 16 | MGI:1098280 | Crebbp | 16 | activation of CREB-binding protein and inhibition of MAPK has a role in cAMP-dependent protein kinase type I regulation of ethanol-induced cAMP response element-mediated gene expression (human) |
| 17 | MGI:97603 | Pkd1 | N/A |  |
| 18 | MGI:97323 | Ngfr | 17 | Data show that sustained activation of p38(MAPK) is essential for the death cascade following exposure of Ewing's sarcoma tumor cells to bFGF and provide evidence that activation of p38(MAPK) results in an up-regulation of the death receptor p75(NTR) (human). |
| 19 | MGI:88388 | Cftr | 18 | A single allelic CFTR mutation is sufficient to augment IL-8 secretion in response to LPS. This is not a result of increased LPS receptor expression but, rather, is associated with alterations in MAPK signaling (human). |
| 20 | MGI:104311 | Ptger4 | 19 | We concluded that PGE2 stimulates the BNP promoter mainly via EP4, PKA, Rap, and p42/44 MAPK (human) |
| 21 | MGI:1100846 | Pparbp | N/A |  |
| 22 | MGI:96748 | Lamp2 | N/A |  |
| 23 | MGI:105923 | Ednra | N/A |  |
| 24 | MGI:98214 | Rxra | N/A |  |
| 25 | MGI:97603 | Pkd1 | N/A |  |
| 26 | MGI:1859631 | Pdgfc | N/A |  |

*References:*

1. Ambrosino, C. et al. TEF-1 and C/EBPbeta are major p38alpha MAPK-regulated transcription factors in proliferating cardiomyocytes. *Biochem J* **396**, 163-72 (2006).

2. Tang, Q.Q. et al. Sequential phosphorylation of CCAAT enhancer-binding protein beta by MAPK and glycogen synthase kinase 3beta is required for adipogenesis. *Proc Natl Acad Sci U S A* **102**, 9766-71 (2005).

3. Bannach, F.G., Gutierrez-Fernandez, A., Parmer, R.J. & Miles, L.A. Interleukin-6-induced plasminogen gene expression in murine hepatocytes is mediated by transcription factor CCAAT/enhancer binding protein beta (C/EBPbeta). *J Thromb Haemost* **2**, 2205-12 (2004).

4. Leu, J.I., Crissey, M.A., Craig, L.E. & Taub, R. Impaired hepatocyte DNA synthetic response posthepatectomy in insulin-like growth factor binding protein 1-deficient mice with defects in C/EBP beta and mitogen-activated protein kinase/extracellular signal-regulated kinase regulation. *Mol Cell Biol* **23**, 1251-9 (2003).

5. Munshi, H.G. et al. Differential regulation of membrane type 1-matrix metalloproteinase activity by ERK 1/2- and p38 MAPK-modulated tissue inhibitor of metalloproteinases 2 expression controls transforming growth factor-beta1-induced pericellular collagenolysis. *J Biol Chem* **279**, 39042-50 (2004).

6. Ingram, D.A. et al. Genetic and biochemical evidence that haploinsufficiency of the Nf1 tumor suppressor gene modulates melanocyte and mast cell fates in vivo. *J Exp Med* **191**, 181-8 (2000).

7. Higuchi, T. et al. MUC20 suppresses the hepatocyte growth factor-induced Grb2-Ras pathway by binding to a multifunctional docking site of met. *Mol Cell Biol* **24**, 7456-68 (2004).

8. Frossi, B., Rivera, J., Hirsch, E. & Pucillo, C. Selective activation of Fyn/PI3K and p38 MAPK regulates IL-4 production in BMMC under nontoxic stress condition. *J Immunol* **178**, 2549-55 (2007).

9. Lovatt, M. et al. Lck regulates the threshold of activation in primary T cells, while both Lck and Fyn contribute to the magnitude of the extracellular signal-related kinase response. *Mol Cell Biol* **26**, 8655-65 (2006).

10. Zhang, X. et al. The HBP1 transcriptional repressor participates in RAS-induced premature senescence. *Mol Cell Biol* **26**, 8252-66 (2006).

11. Wiemann, S. et al. From ORFeome to biology: a functional genomics pipeline. *Genome Res* **14**, 2136-44 (2004).

12. Matthews, L.R. et al. Identification of potential interaction networks using sequence-based searches for conserved protein-protein interactions or "interologs". *Genome Res* **11**, 2120-6 (2001).

13. Hansen, J.B., Petersen, R.K., Jorgensen, C. & Kristiansen, K. Deregulated MAPK activity prevents adipocyte differentiation of fibroblasts lacking the retinoblastoma protein. *J Biol Chem* **277**, 26335-9 (2002).

14. Ulfhammer, E. et al. TNF-alpha mediated suppression of tissue type plasminogen activator expression in vascular endothelial cells is NF-kappaB- and p38 MAPK-dependent. *J Thromb Haemost* **4**, 1781-9 (2006).

15. Medina, M.G. et al. Tissue plasminogen activator mediates amyloid-induced neurotoxicity via Erk1/2 activation. *Embo J* **24**, 1706-16 (2005).

16. Constantinescu, A., Wu, M., Asher, O. & Diamond, I. cAMP-dependent protein kinase type I regulates ethanol-induced cAMP response element-mediated gene expression via activation of CREB-binding protein and inhibition of MAPK. *J Biol Chem* **279**, 43321-9 (2004).

17. Williamson, A.J., Dibling, B.C., Boyne, J.R., Selby, P. & Burchill, S.A. Basic fibroblast growth factor-induced cell death is effected through sustained activation of p38MAPK and up-regulation of the death receptor p75NTR. *J Biol Chem* **279**, 47912-28 (2004).

18. Zaman, M.M. et al. Interleukin 8 secretion from monocytes of subjects heterozygous for the deltaF508 cystic fibrosis transmembrane conductance regulator gene mutation is altered. *Clin Diagn Lab Immunol* **11**, 819-24 (2004).

19. Qian, J.Y., Leung, A., Harding, P. & LaPointe, M.C. PGE2 stimulates human brain natriuretic peptide expression via EP4 and p42/44 MAPK. *Am J Physiol Heart Circ Physiol* **290**, H1740-6 (2006).
